# Methyl viologen-induced changes in the Arabidopsis proteome implicate PATELLIN 4 in oxidative stress responses

**DOI:** 10.1093/jxb/erad363

**Published:** 2023-09-20

**Authors:** Pavol Melicher, Petr Dvořák, Jan Řehák, Olga Šamajová, Tibor Pechan, Jozef Šamaj, Tomáš Takáč

**Affiliations:** Department of Biotechnology, Faculty of Science, Palacký University Olomouc, Olomouc, Czech Republic; Department of Biotechnology, Faculty of Science, Palacký University Olomouc, Olomouc, Czech Republic; Department of Biotechnology, Faculty of Science, Palacký University Olomouc, Olomouc, Czech Republic; Department of Biotechnology, Faculty of Science, Palacký University Olomouc, Olomouc, Czech Republic; Institute for Genomics, Biocomputing and Biotechnology, Mississippi Agricultural and Forestry Experiment Station, Mississippi State University, Starkville, MS, USA; Department of Biotechnology, Faculty of Science, Palacký University Olomouc, Olomouc, Czech Republic; Department of Biotechnology, Faculty of Science, Palacký University Olomouc, Olomouc, Czech Republic; University of Birmingham, UK

**Keywords:** Arabidopsis, chloroplast, IRON SUPEROXIDE DISMUTASE 1, methyl viologen, oxidative stress, PATELLIN 4, photosynthesis, plasma membrane, proteomics

## Abstract

The photosynthesis-induced accumulation of reactive oxygen species in chloroplasts can lead to oxidative stress, triggering changes in protein synthesis, degradation, and the assembly/disassembly of protein complexes. Using shot-gun proteomics, we identified methyl viologen-induced changes in protein abundance in wild-type Arabidopsis and oxidative stress-hypersensitive *fsd1-1* and *fsd1-2* knockout mutants, which are deficient in IRON SUPEROXIDE DISMUTASE 1 (FSD1). The levels of proteins that are localized in chloroplasts and the cytoplasm were modified in all lines treated with methyl viologen. Compared with the wild-type, *fsd1* mutants showed significant changes in metabolic protein and chloroplast chaperone levels, together with increased ratio of cytoplasmic, peroxisomal, and mitochondrial proteins. Different responses in proteins involved in the disassembly of photosystem II–light harvesting chlorophyll *a*/*b* binding proteins were observed. Moreover, the abundance of PATELLIN 4, a phospholipid-binding protein enriched in stomatal lineage, was decreased in response to methyl viologen. Reverse genetic studies using *patl4* knockout mutants and a PATELLIN 4 complemented line indicate that PATELLIN 4 affects plant responses to oxidative stress by effects on stomatal closure.

## Teaser

Arabidopsis rapidly responds to methyl viologen-induced oxidative stress by proteome remodeling. Patellin 4, a plasma membrane protein, likely affects Arabidopsis response to oxidative stress by involvement in stomatal movements.

## Introduction

Plants must adopt an optimal strategy to cope with an unfavorable external environment. The physiological and developmental adaptations require efficient control of transcription; post-transcriptional regulation; protein synthesis, processing, and degradation; post-translational protein modifications; and protein–protein interactions (Huang *et al.*, 2019; Yu *et al.*, 2020; Melicher *et al.*, 2022b). Alteration in the photosynthetic electron transport chain during adverse external conditions leads to an array of complex preventive, protective, and repair mechanisms, including fast protein turnover of photosystem II (PSII) components (Li *et al.*, 2018). Damaged proteins are subject to protein degradation by metalloproteases in chloroplasts and the proteasome complex and to autophagy in the cytosol, and are replaced by *de novo* synthesis (Xiong *et al.*, 2007). This fast protein recycling preconditions the effective photoprotection and plant tolerance to high light and other stresses that affect the photosynthetic apparatus.

D1 is a PSII core protein showing high-speed turnover rates involving rapid degradation mediated by FtsH metalloproteases, fast protein synthesis, and subsequent incorporation of the newly synthesized D1 into PSII (Inagaki, 2022). Other proteins showing degradation rates similar to D1 include protein POST-ILLUMINATION CHLOROPHYLL FLUORESCENCE INCREASE (PIFI; an auxiliary subunit of the NAD(P)H DEHYDROGENASE (NDH) complex; Wang and Portis, 2007; Li *et al.*, 2017), and CYTOCHROME *b*_6_*f* (Cyt *b*_6_*f*) COMPLEX SUBUNIT 4 (PetD), a scaffold protein and plastoquinone binding site component of the Cytochrome *b*_6_*f* complex (Cramer and Zhang, 2006; Li *et al.*, 2017). Prolonged photoinhibition also alters protein profiles in other cellular compartments, including cytosol, mitochondria, and peroxisomes (Taylor *et al.*, 2009; Melicher *et al.*, 2022a). The signal generated by altered redox homeostasis in chloroplasts is transduced to other organelles and compartments by diverse biomolecules such as H_2_O_2_, tetrapyrrols, oxidized carotenoids, and lipids during retrograde signaling (Sierla *et al.*, 2013; Gollan *et al.*, 2015; Chan *et al*., 2016; Exposito-Rodriguez *et al*., 2017) or by redox sensing hubs such as thioredoxins (Dietz and Hell, 2015).

Proteomic and redox proteomic analyses have brought novel insight into the dynamics of protein abundance during photoinhibition or methyl viologen (MV)-induced inhibition of the photosynthetic electron transport chain. Exposure of *Chlamydomonas reinhardtii* cell culture to 0.66 µM MV for 6 h affects the abundance of PSI and PSII subunits, light-harvesting complex proteins, enzymes of the Calvin cycle, and antioxidant enzymes (Nestler *et al.*, 2012). Another highly affected group of proteins is involved in protein turnover, mostly in protein synthesis and degradation (Nestler *et al.*, 2012). In *Chlorella vulgaris*, 72 h of exposure to MV leads to a significant decrease in PsaB and RbcL transcript and protein abundance (Qian *et al.*, 2009). Our recent comparative proteomics study of wild-type (WT) and *fsd1* mutants deficient in IRON SUPEROXIDE DISMUTASE 1 (FSD1) showed that 8 h of MV treatment affected the proteins containing Fe–S clusters as well as components of both photosystems. Fe–S cluster proteins and PSI components were more affected in *fsd1* mutants than WT, showing the importance of antioxidant defense for maintaining proteome homeostasis (Melicher *et al.*, 2022a).

The early response of plants to MV treatment is linked to changes in the profile of oxidation-sensitive proteins. Thus, antioxidant and redox buffering enzymes and regulators of PSII oxygen-evolving complex (OEC) such as PsbO-1, PsbP-1, and PsbQ were identified after 30 min of MV treatment in Arabidopsis (Muthuramalingam *et al.*, 2013). However, short, 30 min MV treatment may lead also to modulation of protein abundance. For example, cytosolic ASCORBATE PEROXIDASE (cAPX) significantly increased in abundance in WT, but not in *fsd1* mutant (Melicher *et al.*, 2022a).

Recently, we characterized the effects of MV on Arabidopsis WT and *fsd1* mutants (Melicher *et al.*, 2022a). MV caused an immediate decrease in PSII activity, which was more pronounced in two *fsd1* mutants. The ascorbate concentration and the level of protein carbonylation were similar to the control after 1 h of MV treatment of WT and mutant plants. Moreover, overactivation of FSD1 and APX was encountered after 30 min MV treatment in Arabidopsis WT, but was not observed in *fsd1* mutants. Thus, short-term MV treatment alters the PSII activity and antioxidant capacity in WT plants (Melicher *et al.*, 2022a).

Early proteome remodeling has not yet been studied in response to MV in plants. Therefore, in this study, we attempted to examine early proteome remodeling associated with MV-induced reactive oxygen species (ROS) accumulation in WT and oxidative stress-sensitive *fsd1* mutants. Our analyses revealed that chloroplastic proteins showed the most prominent sensitivity to MV in WT and *fsd1* mutants. The FSD1 deficiency resulted in proteome remodeling by vigorous change in abundance in metabolic proteins, chloroplastic chaperones, and heat shock proteins. PATELLIN 4 (PATL4), a phospholipid-binding protein localized to plasma membrane, was detected as a protein quickly responding to MV treatment and affecting the oxidative stress response of Arabidopsis. Microscopic analyses showed that PATL4 is involved in the regulation of stomatal aperture during oxidative stress.

## Materials and methods

### Plant material

Arabidopsis WT plants, ecotype Columbia (Col-0), and *fsd1-1*, *fsd1-2* (Dvořák *et al.*, 2021), *patl4-1* (Tejos *et al.*, 2018), and *patl4-2* (GK-091H07, Gabi-Kat Arabidopsis mutant database) mutants, and *patl4-1* mutant expressing *proPATL4::GFP:PATL4* (GFP-PATL4; Tejos *et al.*, 2018) were used in this study. Seed sterilization and *in vitro* plant cultivation on half-strength Murashige–Skoog (½ MS; Murashige and Skoog, 1962) medium were carried out as described in Melicher *et al.* (2022a).

### Proteome investigation

For proteomic analysis, 12-day-old WT, *fsd1-1*, and *fsd1-2* plants were incubated for 30 min in liquid ½ MS medium supplemented with 1 µM MV under 180 μmol m^–2^ s^–1^ illumination. For mock control, plants were incubated in liquid ½ MS medium without MV under the same cultivation conditions. The analysis was performed in four biological replicates, while five whole plants were pooled into one replicate.

Protein extraction, in-solution trypsin digestion, and peptide purification were performed as described in Takáč *et al.* (2017). Briefly, proteins were extracted using phenol followed by protein precipitation in ammonium acetate–methanol. Proteins were dissolved in 6 M urea and digested in-solution using trypsin. Peptides were pre-cleaned on C18 gravitational cartridges (Bond Elut C18; Agilent Technologies, Santa Clara, CA, USA). Nano-LC-MS/MS, protein identification, and protein label-free quantification were performed as in Takáč *et al.* (2021) with modification—intact peptides were measured in the Orbitrap mass detector of the LTQ-Orbitrap hybrid mass spectrometer (Thermo Scientific), and consequently the precursor mass tolerance parameter was narrowed to 10 ppm, when the raw mass spectrometry data were interrogated by Proteome Discoverer 2.1 software (Thermo Scientific).

The differentially abundant proteins were selected based on the criteria ANOVA *P* < 0.05 and fold change >1.5. Proteins present in at least three out of four replicates corresponding to the control proteome and absent in all four replicates of the test proteome were considered as unique for the control proteome, and vice versa.

### Microscopy analysis

To observe the effects of MV on green fluorescent protein (GFP)–PATL4 localization, 4-day-old seedlings were transferred to a microscope slide (containing a spacer from double-sided sticky tape) into liquid ½ MS medium and covered with a coverslip. After initial microscopic observation, plants were treated with 1 µM MV (diluted in ½ MS medium) by perfusion directly at the microscope stage, incubated for 30 min in light (180 μmol m^–2^ s^–1^) or dark (mock control), and documented. Image acquisition was performed using a confocal laser scanning microscope LSM 710 (Carl Zeiss, Jena, Germany) with a Plan-Apochromat ×20/0.8 M27 or alpha Plan-Apochromat ×63/1.46 Oil Korr M27 objective. GFP signal was excited by a 488 nm excitation laser and detected using BP420-480+BP495-550 emission filters. The image acquisition, post-processing, and fluorescence intensity measurement were performed using Zeiss ZEN software (Black and Blue versions, Carl Zeiss). Semi-quantitative evaluation of GFP–PATL4 fluorescence intensity was performed using profile measurement of petiole and leaf epidermal plasma membranes. Data shown as relative fluorescence intensities are obtained from petiole and leaf epidermal cells from five to six individual plants per treatment. Statistical significance was evaluated using Student’s *t-*test. Images of GFP–PATL4 epidermal cells of developing first leaves were processed as orthogonal projections of acquired *Z*-stacks.

### Stomatal aperture and stomatal size measurement

Cotyledons of 4-day-old WT, *patl4-1*, and *patl4-2* were used for stomatal aperture analysis under MV-induced oxidative stress. At first, seedlings were incubated for 30 min in liquid ½ MS medium supplemented with 1 µM MV or without MV as mock control and incubated under 180 μmol m^–2^ s^–1^ illumination. Afterwards, plants were transferred to a microscope slide and into liquid ½ MS medium, covered with a coverslip, and immediately documented using a confocal laser scanning microscope LSM 710 (Carl Zeiss, Jena, Germany) with Plan-Apochromat ×40/1.4 Oil DIC M27 objective in bright field conditions.

To measure stomatal size, cotyledons of 4-day-old WT, *patl4-1*, and *patl4-2* seedlings were incubated in fixative chlorophyll-removing solution (ethanol, acetic acid and glycerol in ratio 3:1:1), which was replaced until the cotyledons were completely discolored. Afterwards, cotyledons were washed in a mixture of ethanol and glycerol (4:1), loaded on a glass slide in a drop of glycerol, covered with a coverslip and analysed with an epifluorescence microscope (Axio Imager.M2) using an EC Plan-Neofluar ×40/0.75 M27 objective in bright field conditions.

The image acquisition, post-processing, stomatal aperture, and stomatal size measurements were performed using Zeiss ZEN software (Black and Blue versions). Stomatal aperture was measured as a ratio of aperture width and length, which is reduced upon closure. Stomatal size was measured as stomatal complex (guard cell pair) length multiplied by its width (Drake *et al.*, 2013). The statistical significance was evaluated by one-way ANOVA with post-hoc Tukey honestly significant difference (HSD) test available at an online web statistical calculator (https://astatsa.com/OneWay_Anova_with_TukeyHSD/).

### Immunoblotting analysis

The abundance of catalase in response to MV treatment was tested in WT, *fsd1-1*, and *fsd1-2* mutants grown and treated as for proteomic analysis. Frozen powdered plant material (100 mg) was resuspended and homogenized with 200 μl extraction buffer (50 mM HEPES, pH 7.5, 75 mM NaCl, 1 mM EGTA, 1 mM MgCl_2_, 1 mM NaF, 1 mM dithiothreitol, 10% glycerol, and Complete™ EDTA-free Protease Inhibitor Cocktail, PhosSTOP™ (Roche Diagnostics, Mannheim, Germany). After 30 min incubation on ice, the extract was centrifuged at 13 000 *g* for 20 min at 4°C. The immunoblotting analysis was carried out using protein extract with 20 µg of total protein as described in Melicher *et al.* (2022a). Catalase (CAT) abundance was detected using anti-CAT primary antibody (AS09 501; Agrisera, Sweden, diluted 1:1000), recognizing all three catalase isoforms in Arabidopsis. The protein abundance was expressed as an average of band optical densities from three biological replicates. The statistical significance was evaluated by one-way ANOVA with post-hoc Tukey HSD test.

### Phenotype and chlorophyll content analysis

The fresh weight of 15–19 seedlings of 14-day-old *patl4* mutants grown *in vitro* was recorded per line and replicate. Three biological replicates were performed and the statistical significance was evaluated by one-way ANOVA with post-hoc Tukey HSD test. Seven- and 11-day-old plants were documented for comparison using a flat scanner (ImageScanner III, GE Healthcare, UK).

To examine plant phenotypic responses to MV, 5-day-old seedlings of WT, *fsd1-1*, *patl4* mutants, and the GFP-PATL4 line growing on ½ MS medium were transferred to ½ MS medium with or without 2 µM MV. All measurements were performed in three repetitions. Seedlings were documented on the seventh day after the transfer, and the tolerance to MV was expressed as the percentage of green plants from 50 seedlings per replicate (150 in total). In addition, the primary root length of 30 MV-treated seedlings per replicate (90 in total) and 20 control seedlings per replicate (60 in total) was measured using ImageJ software (Schneider *et al.*, 2012) on the seventh day after the seedling transfer. Statistical significance was evaluated by one-way ANOVA with post-hoc Tukey HSD test.

The relative amount of chlorophyll *a* and *b* was measured according to Barnes *et al.* (1992). The concentrations of chlorophyll *a* and *b* were measured using 30 seedlings for each line and the measurements were expressed per fresh weight of examined seedlings performed in triplicate (90 seedlings examined in total). The statistical significance was evaluated by one-way ANOVA with post-hoc Tukey HSD test.

## Results

### Comparative shot-gun proteomic analysis

In this study, shot-gun quantitative proteomics revealed that 9, 11, and 19 proteins showed statistically significant changes in abundance in MV-treated as compared with mock-treated WT, *fsd1-1*, and *fsd1-2* plants, respectively (Supplementary Tables S1–S3). Several other proteins showed unique occurrences in either mock- or MV-treated samples. We consider proteins identified only in mock-treated controls as down-regulated in MV-treated samples, as they were below the detection threshold of the instrument. In accordance, proteins identified in MV-treated samples but not in mock-treated ones are considered up-regulated in MV-treated plants. Therefore, the uniquely identified proteins (14, 7, and 33 proteins in WT, *fsd1-1*, and *fsd1-2* mutant, respectively) were evaluated together with the quantitatively identified differentially abundant proteins (Supplementary Tables S1–S3).

**Fig. 1.**
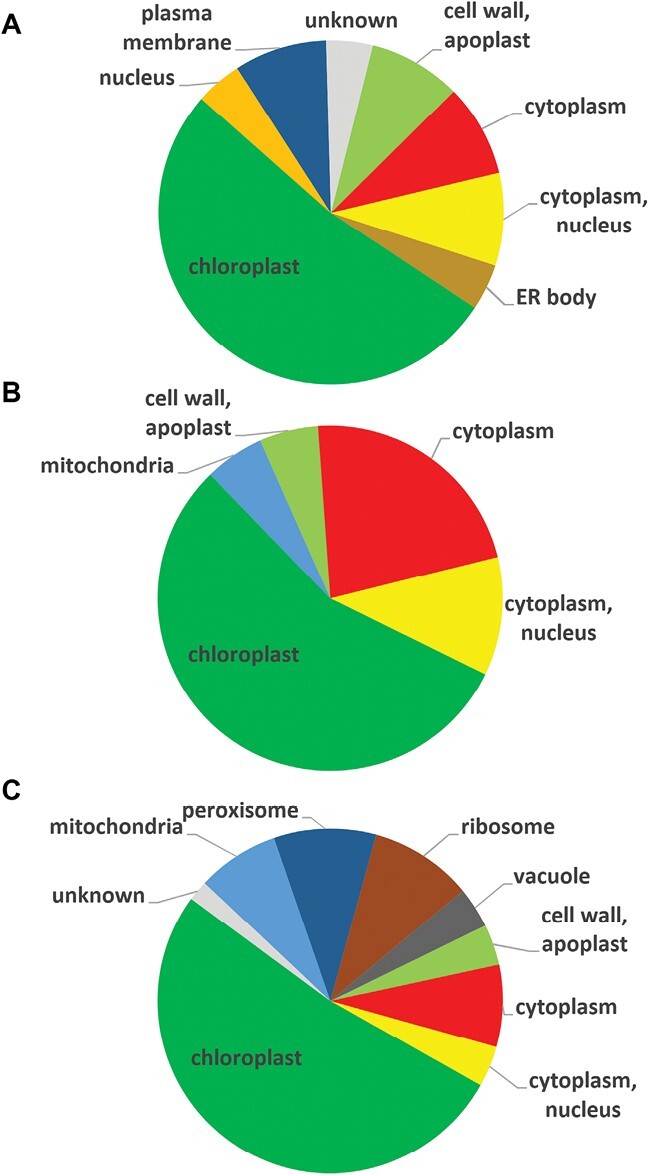
Comparison of the distribution of differentially regulated proteins in subcellular compartments found in Col-0 wild-type plants (A), *fsd1-1* (B), and *fsd1-2* mutants (C) after 30 min methyl viologen treatment. ER, endoplasmic reticulum.

**Fig. 2.**
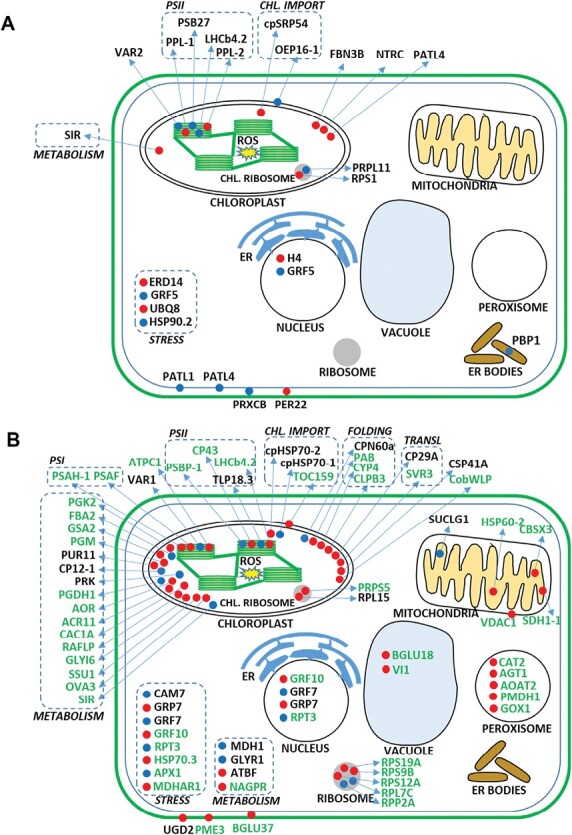
Schematic representation of the differential proteomes of Col-0 wild-type (A) and *fsd1-1* and *fsd1-2* mutant (B) plants exposed to 30 min methyl viologen treatment. Up-regulated and down-regulated proteins are depicted as red and blue circles, respectively. In (B), the abbreviations of differentially regulated proteins found in *fsd1-1* mutant are shown in black and those found in *fsd1-2* mutant are shown in green. Proteins with similar functions are framed by a dashed line. Abbreviations: ACR11, uridylyltransferase-like protein; AGT1, alanine:glyoxylate aminotransferase; AOAT2, alanine-2-oxoglutarate aminotransferase 2; AOR, oxidoreductase zinc-binding dehydrogenase family protein; APX1, ascorbate peroxidase 1; ATBF, aldolase-type TIM barrel family protein; AtGLYI6, glyoxalase/Bleomycin resistance protein/Dioxygenase superfamily protein (GLYOXALASE I6); AtNAGPR, putative *N*-acetyl-γ-glutamyl-phosphate reductase; AtOEP16-1, outer plastid envelope protein 16-1; ATPC1, ATPase F_1_ complex γ subunit protein; AtPRK, phosphoribulokinase; AtPUR11, adenylosuccinate synthase; AtSIR, sulfite reductase; BGLU18, β-glucosidase 18; BGLU37, glucoside glucohydrolase 2 (β-glucosidase 37); CAC1A, chloroplastic acetylcoenzyme A carboxylase 1; CAM7, calmodulin 7; CAT2, catalase 2; CBSX3, cystathionine β-synthase (CBS) family protein; CLPB3, casein lytic proteinase B3; CobWLP, cobalamin biosynthesis CobW-like protein; CP12-1, CP12 domain-containing protein 1; CP29A, chloroplast RNA-binding protein 29; CP43, photosystem II 44 kDa protein; cpHSP70, chloroplast heat shock protein 70-1; cpHSP70-2, chloroplast heat shock protein 70-2; CPN60a, chaperonin-60α; cpSRP54, chloroplast signal recognition particle 54 kDa subunit; CSP41A, chloroplast stem-loop binding protein of 41 kDa; CYP4, rotamase CYP 4; ER, endoplasmic reticulum; ERD14, dehydrin family protein; FBA2, fructose-bisphosphate aldolase 2; FBN3B, plastid-lipid associated protein PAP/fibrillin family protein; GFR10, general regulatory factor 10; GLYR1, glyoxylate reductase 1; GOX1, aldolase-type TIM barrel family protein; GRF5, general regulatory factor 5; GRF7, general regulatory factor 7; GRP7, cold circadian rhythm and RNA binding 2; GSA2, glutamate-1-semialdehyde 2.1-aminomutase 2; H4, histone superfamily protein; HSP60-2, heat shock protein 60-2; HSP70.3, heat shock protein 70-3; HSP90.2, heat shock protein 81-2; Lhcb4.2, light harvesting complex photosystem II; MDH1, lactate/malate dehydrogenase family protein; MDHAR1, monodehydroascorbate reductase 1; NTRC, NADPH-dependent thioredoxin reductase C; nuclear protein; OVA3, glutamate tRNA synthetase; PAB, cyclin delta-3 (protein in chloroplast ATPase biogenesis); PATL1, patellin 1; PATL4, patellin 4; PBP1, PYK10-binding protein 1; PER22, peroxidase superfamily protein; PGDH1, d-3-phosphoglycerate dehydrogenase; PGK2, phosphoglycerate kinase family protein; PGM, phosphoglucomutase putative/glucose phosphomutase; PMDH1, peroxisomal NAD-malate dehydrogenase 1; PME3, pectin methylesterase 3; PPL-1, PsbP-like protein 1; PPL-2, PsbP-like protein 2; PRPL11, plastid ribosomal protein l11; PRPS5, ribosomal protein S5 family protein; PRXCB, peroxidase CB; PsaF, photosystem I subunit F; PsaH-1, photosystem I subunit H-1; Psb27, photosystem II family protein; PsbP-1, photosystem II subunit P-1; RAFLP, Rubisco accumulation factor-like protein; RPL15, ribosomal protein L15; RPL7C, ribosomal protein L30/L7 family protein; RPP2A, 60S acidic ribosomal protein family; RPS1, ribosomal protein S1; RPS12A, ribosomal protein L7Ae/L30e/S12e/Gadd45 family protein; RPS19A, ribosomal protein S19e family protein; RPS9B, ribosomal protein S4; RPT3, regulatory particle triple-A ATPase 3; SDH1-1, succinate dehydrogenase 1-1; SIR, sulfite reductase; SSU1, aconitase/3-isopropylmalate dehydratase protein; SUCLG1, succinyl-CoA ligase α-subunit; SVR3, elongation factor family protein; TLP18.3, thylakoid lumen 18.3 kDa protein; TOC159, translocon at the outer envelope membrane of chloroplasts 159; TRA2, aldolase-type TIM barrel family protein; UBQ8, ubiquitin 8; UGD2, UDP-glucose 6-dehydrogenase family protein; VAR1, FtsH extracellular protease family; VAR2, FtsH extracellular protease family; VDAC1, voltage-dependent anion channel 1; VI1, glycosyl hydrolases family 32 protein (vacuolar invertase 1).

More than half of the MV-affected proteins were localized to the chloroplast in all three lines (Fig. 1). The number of cytoplasmic proteins was higher in the mutants compared with WT. Unlike mutants, MV affected two plasma membrane-localized proteins (PATL1 and PATL4) and two apoplastic peroxidases in WT. On the other hand, MV caused deregulation of mitochondrial and peroxisomal proteins in the mutants but not in WT plants (Figs 1, 2).

The profile of differentially regulated chloroplastic proteins differed in mutants and WT (Fig. 2). In WT plants, MV dramatically affected the PsbP-LIKE PROTEIN 1 (PPL-1), an extrinsic subunit of PSII and a PHOTOSYSTEM II FAMILY PROTEIN Psb27 localized in the thylakoid lumen. While PPL-1 showed substantial, 6.5-fold up-regulation, the abundance of Psb47 was considerably down-regulated (0.11-fold; Table 1). MV also negatively affected the LIGHT-HARVESTING COMPLEX PHOTOSYSTEM II Lhcb4.2, which was detected only in mock-treated WT samples. A component of the chloroplastic NDH complex, a PsbP-LIKE PROTEIN 2 (PPL-2) was uniquely found only in MV-treated WT plants.

**Table 1.** Proteins involved in photosynthesis and differentially abundant in WT, *fsd1-1*, and *fsd1-2* mutants after 30 min methyl viologen (MV) treatment

	Abundance ratio (MV treatment versus mock treatment)			
Protein	Col-0	*fsd1-1*	*fsd1-2*	Function and Reference
Photosystem complex components				
Photosystem II family protein	0.12			Psb27; thylakoid lumen protein; required for PSII repair (Chen *et al.*, 2006); facilitates D1 protein precursor processing during PSII biogenesis (Wei *et al.*, 2010)
PsbP-like protein 1	6.56			Extrinsic subunit of photosystem II; facilitates the assembly of the photosystem II supercomplexes (Che *et al.*, 2020)
PsbP-like protein 2	Unique in MV-treated sample			Component of the lumenal subcomplex L of chloroplast NDH complex, required for accumulation of the NDH complex (Ishihara *et al.*, 2007; Ifuku *et al.*, 2011)
Photosystem II subunit P-1			0.30	Photosystem II extrinsic subunit, water splitting and oxygen evolution (Ifuku, 2014)
Photosystem II 44 kDa protein (CP43)			Unique in MV-treated sample	Photosystem II core antenna protein
Light harvesting complex photosystem II	Unique in mock control		Unique in MV-treated sample	Lhcb4.2; important for photoprotection (de Bianchi *et al.*, 2011)
Photosystem I subunit F			2.07	Photosystem I component
Photosystem I subunit H-1			Unique in MV-treated sample	Photosystem I component
Photoprotection				
FtsH extracellular protease family VAR2	0.32			VAR1 and VAR2; protease involved in D1 degradation and repair cycle (Takechi *et al.*, 2000)
FtsH extracellular protease family VAR1		Unique in mock control		VAR1 and VAR2; protease involved in D1 degradation and repair cycle (Takechi *et al.*, 2000)
Chloroplast RNA-binding protein 29		Unique in MV-treated sample		High light induced dissassembly of PSII supercomplexes (Fristedt and Vener, 2011)
Outer plastid envelope protein 16-1	Unique in mock control			Photoprotective role by preventing singlet oxygen production (Buhr *et al.*, 2008)
Fibrillin family protein FBN2	Unique in MV-treated sample			Important for PSII protection (Torres-Romero *et al*, 2021)
Thylakoid lumen 18.3 kDa protein		0.43		PSII repair cycle (Järvi *et al.*, 2016)
Plastid protein import and processing				
Translocon at the outer envelope membrane of chloroplasts 159			Unique in MV-treated sample	Plastid protein import
Chloroplast heat shock protein 70-1			0.38	Plastid protein import (Su and Li, 2010)
Chloroplast heat shock protein 70-2		2.65		Plastid protein import (Su and Li, 2010)
Chloroplast signal recognition particle 54 kDa subunit	Unique in MV-treated sample			Co-translational and post-translational sorting of thylakoid proteins (Rutschow *et al*., 2008)
Chaperonin-60α		0.55		Protein folding
Cyclin δ3			Unique in MV-treated sample	Assembly chaperone of chloroplast ATP synthase coupling factor 1 (Mao *et al.*, 2015)
Casein lytic proteinase B3			Unique in MV-treated sample	chloroplastic disaggregase (Parcerisa *et al.*, 2020)
Other functions				
Chloroplast stem-loop binding protein of 41 kDa		18.02		Plastid transcript stabilization (Qi *et al.*, 2012)
CP12 domain-containing protein 1		Unique in mock control		Calvin–Benson cycle regulation (López-Calcagno *et al*., 2014)
Chlorophyll A-B binding family protein			Unique in MV-treated sample	Non-photochemical quenching (Steen *et al*., 2020)
Unknown function				
Rubisco accumulation factor-like protein			Unique in MV-treated sample	Unknown

Opposite to WT, Lhcb4.2 was up-regulated in *fsd1-2* mutant. MV also disturbed the abundance of PSII components in this mutant, including PHOTOSYSTEM II SUBUNIT P-1 (0.30-fold down-regulated) and PHOTOSYSTEM II 44 kDa PROTEIN (CP43), showing full homology to PHOTOSYSTEM II CORE ANTENNA PROTEIN C (ATCG00280; uniquely found in the MV-treated *fsd1-2* mutant). In addition to PSII, the *fsd1-2* mutant exhibited changes in PSI subunits. According to quantitative evaluation, PHOTOSYSTEM I SUBUNIT F (PSI-F) was 2-fold up-regulated in the *fsd1-2* mutant and PHOTOSYSTEM I SUBUNIT H-1 (PSI-H1) was uniquely found in *fsd1-2* mock-treated sample. The *fsd1-1* did not show significant changes in photosystem subunit abundances.

We also compared the abundances of proteins between MV-treated mutants with MV-treated WT plants (Supplementary Tables S4, S5). Both mutants had altered abundances of two PSI subunits. While PSI-N showed lower abundance in the mutants, the abundance of PSI-H1 was higher. PSII light harvesting complex protein B1B2 showed lower abundance in the *fsd1-1* mutant.

Furthermore, MV altered the abundance of proteins involved in chloroplast protein import in WT and *fsd1* mutants, while different proteins were affected in these lines (Fig. 2). In WT, MV altered the abundance of OUTER PLASTID ENVELOPE PROTEIN 16-1 and CHLOROPLAST SIGNAL RECOGNITION PARTICLE 54 kDa SUBUNIT, which were detected only in mock and MV-treated WT plants, respectively. In mutants, TRANSLOCON AT THE OUTER ENVELOPE MEMBRANE OF CHLOROPLAST 159 (found only in MV-treated *fsd1-2*), chloroplast HEAT SHOCK PROTEIN (HSP) 70-1 (0.38-fold down-regulated in *fsd1-2*) and chloroplast HSP 70-2 (2.65-fold up-regulated in *fsd1-1*) were affected. Thus, FSD1 deficiency conferred the alteration of chloroplast HSP70 isoforms in response to short-term MV treatment. Notably, *fsd1* mutants exhibited higher alterations in abundances of proteins involved in chloroplast protein folding (Fig. 2), including CHAPERONIN-60 ALPHA (0.55-fold down-regulated in *fsd1-1*), CYCLIN DELTA-3, and CASEIN LYTIC PROTEINASE B3 (both found uniquely in MV-treated *fsd1-2*). Plastid FtsH extracellular protease family protein ATP-DEPENDENT ZINC METALLOPROTEASE FTSH 2 (VAR2), involved in the chloroplastic protein degradation pathway, was 0.31-fold down-regulated in WT. Similarly, *fsd1-1* exhibited down-regulation of ATP-DEPENDENT ZINC METALLOPROTEASE FTSH 5 (VAR1), found only in mock-treated *fsd1-1* plants.

The comparison of MV-treated mutants and WT plants showed altered abundances of proteins involved in chloroplast protein folding in the mutants (Supplementary Tables S4, S5). While CHAPERONIN 20 showed lower abundance in both *fsd1* mutants, ATP-DEPENDENT CASEINOLYTIC (CLP) PROTEASE/CROTONASE FAMILY PROTEIN was more abundant in *fsd1-1*, and ROTAMASE CYP 4 in *fsd1-2*. TRIGGER FACTOR TYPE CHAPERONE FAMILY PROTEIN showed lower abundance in *fsd1-2* mutant. The *fsd1-1* mutant had lower abundance of TRANSLOCON AT THE OUTER ENVELOPE MEMBRANE OF CHLOROPLASTS 159 protein compared with WT. In addition, both mutants showed altered abundances of plastid ribosomal proteins (Supplementary Tables S4, S5).

Abundances of proteins involved in protein folding in cytosol were also altered by MV (Fig. 2). HSP 81-2, also known as HSP90.2, localized in cytosol, was down-regulated in WT plants, while the opposite regulation was found for another HSP isoform, HSP 81-3 (HSP 90.3) in *fsd1-2* mutant. After the MV treatment, the levels of cytoplasmic proteins involved in protein folding did not show significant differences between the mutants and WT (Supplementary Tables S4, S5).

Chloroplastic ribosomal proteins did not differ among the lines, but cytosolic ribosomal proteins were detected only in *fsd1-2* mutant. RIBOSOMAL PROTEIN L7Ae/L30e/S12e/Gadd45 FAMILY PROTEIN (RPS12A), RIBOSOMAL PROTEIN S4 (RPS9B) and RIBOSOMAL PROTEIN S19e FAMILY PROTEIN (RPS19A) were up-regulated, while 60S ACIDIC RIBOSOMAL PROTEIN FAMILY (RPP2A) and RIBOSOMAL PROTEIN L30/L7 FAMILY PROTEIN (RPL7C) were down-regulated (Fig. 2). The levels of cytosolic ribosomal proteins differed in MV-treated *fsd1* mutants from WT (Supplementary Tables S4, S5).

**Fig. 3.**
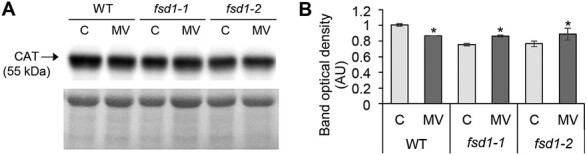
Immunoblotting analysis of CATALASE (CAT) in Col-0 wild-type (WT) plants, *fsd1-1*, and *fsd1-2* mutants subjected to 30 min methyl viologen (MV) treatment. (A) Immunoblot of CAT supplemented with respective control of protein loading visualized by Ponceau S staining. (B) Quantification of band optical densities in (A). Lane C, blot containing protein extracts from mock; lane MV, MV-treated plants. Raw band optical densities were normalized according to the total density of the specific bands on the membrane (mean ±SD, *n*=3). Asterisks indicate a statistically significant difference between mock control and MV treatment as revealed by one-way ANOVA with post-hoc Tukey HSD test (*P*<0.05). Uncropped, full original images of blot and Ponceau S stained membrane are provided in Supplementary Fig. S1.

MV treatment disturbed the abundance of proteins involved in ROS decomposition in *fsd1-2* mutant (Fig. 2), showing up-regulation of cytosolic MONODEHYDROASCORBATE REDUCTASE 1 (2.75-fold) and CATALASE 2 (2.5-fold) and down-regulation of APX1 (0.33-fold). In agreement, immunoblotting analysis using anti-CAT primary antibody recognizing all three Arabidopsis CAT isoforms showed an increase of total CAT abundance in *fsd1* mutants, while a decrease was observed in WT plants (Fig. 3). In WT, we encountered a higher abundance of NADPH-DEPENDENT THIOREDOXIN REDUCTASE C. Nevertheless, the levels of antioxidant enzymes did not differ significantly between *fsd1* mutants and WT after MV treatment (Supplementary Tables S4, S5).

Some other stress-related proteins showed unique deregulation in WT (Fig. 2). Notably, we observed down-regulation of phospholipid binding, plasma membrane-localized PATL1 and PATL4 after MV treatment of WT plants. MV also affected the abundance of cytosolic DEHYDRIN FAMILY PROTEIN (ERD14), showing substantial 4.7-fold up-regulation, and a defense-related protein PYK10-BINDING-PROTEIN 1 localized to endoplasmic reticulum bodies (down-regulated). A nuclear protein, HISTONE SUPERFAMILY PROTEIN (putative H4-TYPE HISTONE), was dramatically up-regulated after MV treatment in WT.

Striking differences between WT and the mutants were found in metabolic proteins (Fig. 2). While negligible effects were found in WT, the metabolic proteins showed substantial abundance changes in both mutants. MV increased the abundance of proteins involved in amino acid biosynthesis, photorespiration, carbohydrate degradation, and detoxification of reactive carbonyls and methylglyoxal. In addition, abundances of proteins involved in mitochondrial electron transport chain, glycolysis, and energy homeostasis were also disturbed (Table 2). After MV treatment, the mutants differed from WT mainly in proteins involved in glycolysis/gluconeogenesis and amino acid biosynthesis (Supplementary Tables S4, S5).

**Table 2.** Proteins involved in metabolic processes and differentially abundant in WT, *fsd1-1*, and *fsd1-2* mutants after 30 min MV treatment

Description	Function	Localization (UniProt)	Abundance ratio (MV treatment versus mock treatment)	Allele
WT				
NADPH-dependent thioredoxin reductase C; NTRC	Redox regulation	Chloroplast	Unique in WT MV	—
Sulfite reductase; SIR	Sulfate assimilation	Chloroplast	Unique in WT MV	—
*fsd1* mutants				
ATPase, F_1_ complex, γ-subunit protein; ATPC1	ATP biosynthesis	Chloroplast	2.54	*fsd1-2*
Phosphoribulokinase; PRK	Calvin cycle	Chloroplast	0.13	*fsd1-1*
CP12 domain-containing protein 1; CP12-1		Chloroplast	Unique in *fsd1* mock	*fsd1-1*
Phosphoglycerate kinase family protein; PGK2		Chloroplast	3.21	*fsd1-2*
Rubisco accumulation factor-like protein; RAFLP		Chloroplast	Unique in *fsd1* MV	*fsd1-2*
Alanine:glyoxylate aminotransferase; AGT1	Photorespiration	Peroxisome	Unique in *fsd1* MV	*fsd1-2*
Aldolase-type TIM barrel family protein; GOX1		Peroxisomes	14.53	*fsd1-2*
Aldolase-type TIM barrel family protein; TRA2	Pentose-phosphate pathway	Unknown	Unique in *fsd1* MV	*fsd1-2*
Succinate dehydrogenase 1-1; SDH1-1	Electron transport chain	Mitochondrion	Unique in *fsd1* MV	*fsd1-2*
Succinyl-CoA ligase, α-subunit; SUCLG1	Tricarboxylic acid cycle	Mitochondria	Unique in *fsd1* mock	*fsd1-1*
Lactate/malate dehydrogenase family protein; MDH1		Cytoplasm	0.13	*fsd1-1*
Fructose-bisphosphate aldolase 2; FBA2	Sugar metabolism	Chloroplast	0.36	*fsd1-2*
Phosphoglucomutase, putative/glucose phosphomutase; PGM		Unknown	Unique in *fsd1* MV	*fsd1-2*
Glyoxalase/Bleomycin resistance protein/Dioxygenase superfamily protein; GLYI6	Detoxification	Chloroplast	Unique in *fsd1* MV	*fsd1-2*
Peroxisomal NAD-malate dehydrogenase 1; PMDH1		Peroxisome	Unique in *fsd1* MV	*fsd1-2*
Oxidoreductase, zinc-binding dehydrogenase family protein; AOR		Chloroplast	2.13	*fsd1-2*
Glyoxylate reductase 1; GLYR1		Cytoplasm	0.30	*fsd1-1*
Adenylosuccinate synthase; PUR11	Nucleotide biosynthesis	Chloroplast	6.56	*fsd1-1*
UDP-glucose 6-dehydrogenase family protein; UGD2		Cell wall	Unique in *fsd1* MV	*fsd1-1*
d-3-Phosphoglycerate dehydrogenase; PGDH1	Amino acid metabolism	Chloroplast	0.4	*fsd1-2*
Uridylyltransferase-like protein; ACR11		Chloroplast	4.40	*fsd1-2*
Aconitase/3-isopropylmalate dehydratase protein; SSU1		Chloroplast	Unique in *fsd1* MV	*fsd1-2*
Putative *N*-acetyl-γ-glutamyl-phosphate reductase; NAGPR		Cytoplasm	Unique in *fsd1* MV	*fsd1-2*
Alanine-2-oxoglutarate aminotransferase 2; AOAT2		Peroxisome	Unique in *fsd1* MV	*fsd1-2*
Glutamate tRNA synthetase; OVA3	Protein synthesis	Chloroplast	Unique in *fsd1* MV	*fsd1-2*
Glucoside glucohydrolase 2; BGLU37	Glucosinolate metabolism	Apoplast	Unique in *fsd1* MV	*fsd1-2*
β-Glucosidase 18; BGLU18		Vacuole	Unique in *fsd1* MV	*fsd1-2*
Glycosyl hydrolases family 32 protein; VI1	Glycan metabolism	Vacuole	Unique in *fsd1* MV	*fsd1-2*
Pectin methylesterase 3; PME3		Cell wall	Unique in *fsd1* MV	*fsd1-2*
Chloroplastic acetylcoenzyme A carboxylase 1; CAC1A	Lipid metabolism	Chloroplast	Unique in *fsd1* MV	*fsd1-2*
Sulfite reductase; SIR	Sulfate assimilation	Chloroplast	Unique in *fsd1* mock	*fsd1-2*
Glutamate-1-semialdehyde 2,1-aminomutase 2; GSA2	Tetrapyrrole biosynthesis	Chloroplast	12.45	*fsd1-2*
Aldolase-type TIM barrel family protein; ATBF	Unknown	Cytoplasm	Unique in *fsd1* MV	*fsd1-1*

### PATL4 abundance drops shortly after methyl viologen treatment in Arabidopsis wild-type

Proteomic analysis indicated down-regulation of PATL4 abundance in response to short-term MV treatment in WT. Patellins are plasma membrane-localized proteins. Their possible involvement in plant oxidative stress has not been described yet. Therefore, the response of PATL4 to MV was evaluated by live-cell confocal laser scanning microscopy in *patl4-1* mutants expressing the *proPATL4::GFP:PATL4* construct.

**Fig. 4.**
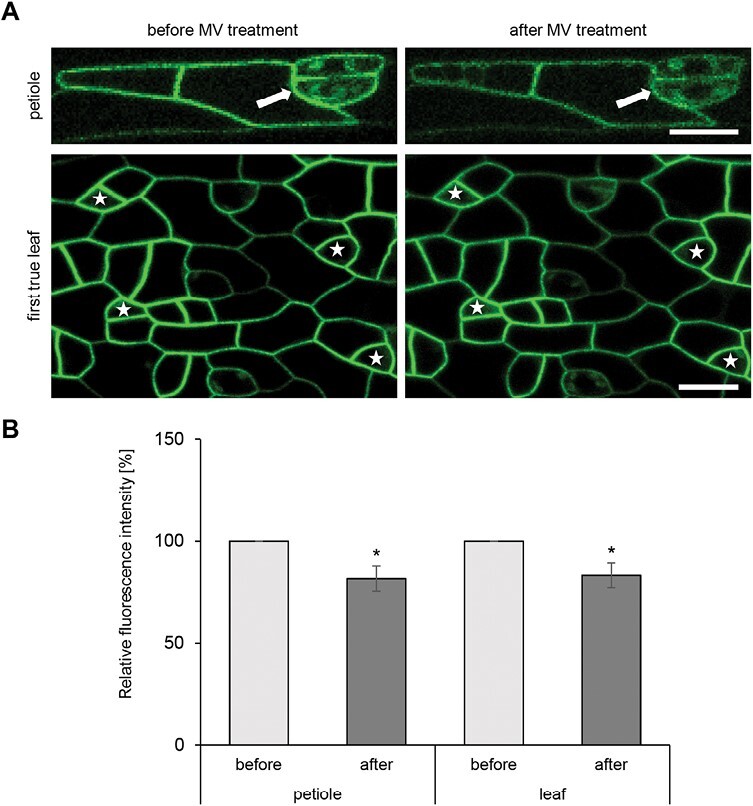
GFP–PTL4 localization in epidermal cells of Arabidopsis petiole and leaf after methyl viologen (MV) treatment revealed by confocal laser scanning microscopy. (A) Plasma membrane localization of GFP–PTL4 in epidermal cells of petiole and first true leaf before and after 30 min treatment with MV in the light. Stomata are indicated by arrows and meristemoids by stars. (B) Semiquantitative analysis of plasma membrane GFP–PTL4 fluorescence intensity of petiole and leaf epidermal cells before and after 30 min treatment with MV in the light. Asterisks indicate statistical significance between treated and not treated cells (*P<0.05). Error bars show ±standard deviation. Scale bar, 10 µm.

We monitored the GFP–PATL4 fluorescence in epidermal cells of petioles and first true leaves. We found a decrease in GFP signal intensity after 30 min MV treatment at the plasma membranes of petiole and leaf epidermal cells (Fig. 4). Control MV treatment carried out in the dark did not affect GFP–PATL4 signal intensity Supplementary Fig. S2).

### Phenotypic analysis of Arabidopsis patl4 mutants exposed to methyl viologen

**Fig. 5.**
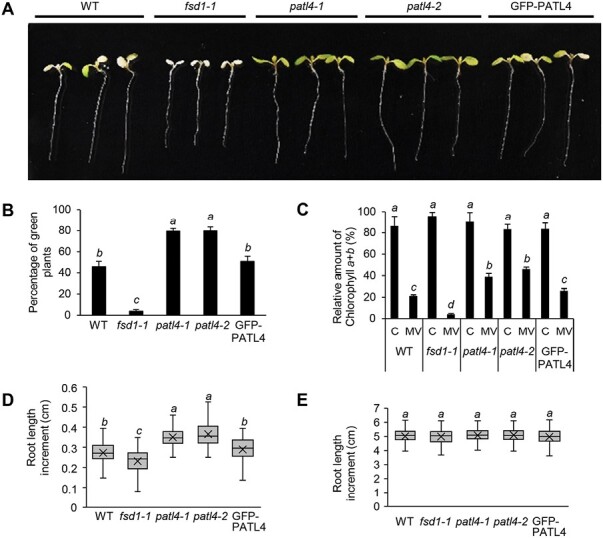
Phenotypic response of Col-0 wild-type (WT), *fsd1-1* mutant, *patl4* mutants, and GFP–PATL4 complemented line to 2 μM methyl viologen (MV). (A) Representative images of seedlings documented on seventh day after the transfer to medium with MV. (B) Quantification of fully green seedlings from all examined seedlings (mean ±SD; 50 seedlings per *n*; *n*=3). (C) Quantification of relative amount of chlorophylls *a* and *b* in plants treated by MV (mean ±SD; *n*=3). (D) Quantification of root growth inhibition in response to MV, expressed as root length increment (30 seedlings per *n*; *n*=3). (E) Quantification of root growth in control conditions expressed as root length increment (20 seedlings per *n*; *n*=3). Statistically significant difference at a *P*<0.05 as determined by one-way ANOVA with post-hoc Tukey HSD test (mean ±SD) indicated by letters above the columns/boxes.

To examine the involvement of PATL4 in plant responses to MV, we transferred 5-day-old seedlings of WT, *fsd1-1*, *patl4* mutants (*patl4-1* and *patl4-2*) and GFP–PATL4 complemented line to medium supplemented with 2 µM MV (Fig. 5).

As expected, *fsd1-1* mutant showed hypersensitivity to MV treatment, exemplified by almost completely bleached cotyledons and the lowest chlorophyll content (Fig. 5A–C). It also showed the highest levels of root growth inhibition (Fig. 5D). On the other hand, both *patl4* mutants show the highest tolerance to MV, indicated by the highest ratio of fully green cotyledons, chlorophyll content (Fig. 5A–C), and the highest increment of root length (Fig. 5D). WT plants and the GFP–PATL4 line showed intermediate phenotypes (Fig. 5A, B) and chlorophyll content (Fig. 5C), supporting that PATL4 complementation reversed phenotype of *patl4* mutant. Under control conditions, the lines did not exhibit significant differences in root growth rate and primary root length (Fig. 5E; Supplementary Fig. S3A, B). However, *patl4* mutants exhibited significantly higher fresh weight compared with WT, apparently due to the bigger size of leaf rosettes (Supplementary Fig. S3B, C). These data indicate that PATL4 affects the plant tolerance to MV-induced oxidative stress.

### Measurement of stomata size and aperture after methyl viologen treatment

**Fig. 6.**
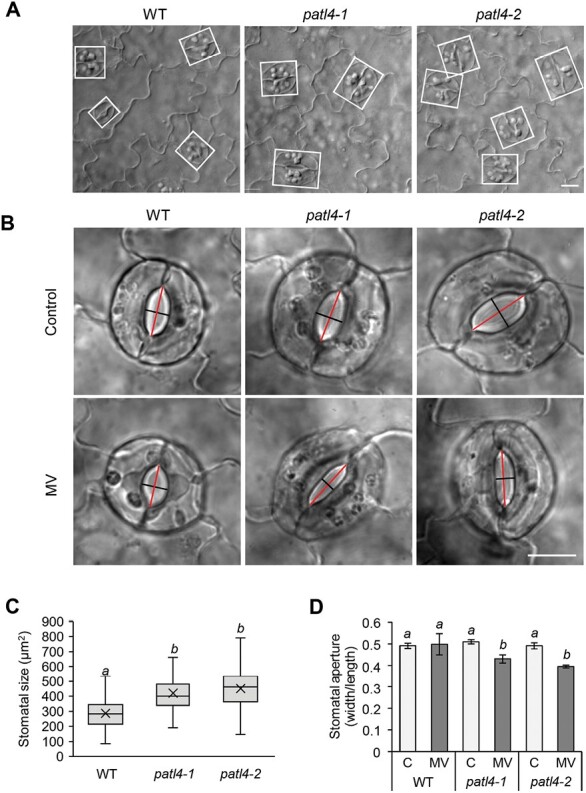
Stomatal size and aperture measurements in *patl4* mutants. (A) Representative images of epidermal cells of cotyledon abaxial sides in wild-type (WT), *patl4-1*, and *patl4-2* mutants under control conditions. (B) Representative images of stomata of WT, *patl4-1*, and *patl4-2* mutants incubated in a liquid half-strength MS medium without (Control) or with 1 µM methyl viologen (MV). (C) Quantification of stomatal size under control conditions. Stomatal size was measured as stomatal width×length as depicted by white rectangles in (A) (*n*=200 stomata). (D) Quantification of stomatal aperture under control (C) and after MV treatment conditions. Stomatal aperture was quantified as a ratio of aperture width to aperture length as depicted by black (width) and red (length) lines in (B) (mean ±SD, 20 stomata per *n*, *n*=3). Statistically significant difference at a *P*<0.05 as determined by one-way ANOVA with post-hoc Tukey HSD test is indicated by letters above the boxes/columns. Scale bar, 10 µm.

The prevalence of GFP–PATL4 in plasma membranes of stomatal precursors and stomata indicated its possible role in stomatal development and/or movement. To examine whether PATL4 may be involved in stomatal movement, we measured stomatal aperture of *patl4* mutants under MV-induced oxidative stress. We found that MV caused more pronounced closure of stomata in *patl4* mutants as confirmed by lower stomatal aperture of *patl4* mutants (Fig. 6A, B). Moreover, the size of stomata in the mutants was significantly larger than in WT (Fig. 6A, C). Thus, PATL4 may have a regulatory role in stomatal development and movement during oxidative stress.

## Discussion

Alterations of protein abundance detected by shot-gun proteomic analysis after stress treatments that last in the range of minutes require careful interpretation. The average protein half-life is estimated to be approximately 3 d and may differ under stress conditions (Nelson *et al.*, 2014). Therefore, changes in abundance may reflect protein synthesis or degradation dynamics, as was most likely in the case of PHOTOSYSTEM II CP43 REACTION CENTER PROTEIN (up-regulated in *fsd1-2* mutant), which undergoes fast repair during photodamage (Kato and Sakamoto, 2009). Alternatively, proteins displaying differential regulation may disassemble from complexes (such as Lhcb4.2; de Bianchi *et al.*, 2011) or release from membranes, causing better accessibility to protein digestion during sample preparation. Many of the identified differentially regulated proteins were previously shown to be involved in photoprotection (Table 1), confirming that compositions of differential proteomes are relevant to oxidative stress generated in chloroplasts. Our approach provides an opportunity to get information on the abundance of proteins localized in diverse subcellular compartments and correlate them with various biochemical pathways. These proteins are likely involved in early molecular mechanisms of plant responses to oxidative stress generated in chloroplasts, while proteins identified in *fsd1* mutants are likely associated with plant hypersensitivity to oxidative stress. Notably, the phenol extraction coupled to methanol precipitation used in this study can extract the hydrophobic proteins embedded in the membranes with lower efficiency.

### fsd1 mutants differ from wild-type in photosystem II–light harvesting complex disassembly, chloroplastic protein folding, and metabolism

The different susceptibility of WT and the *fsd1* mutants to MV-induced oxidative stress was reflected in the composition and the intraorganellar localization of differentially regulated chloroplastic proteins (Fig. 2). The abundance of Lhcb4.2, a component of the light-harvesting complex of photosystem II, was down-regulated in WT but up-regulated in the *fsd1-2* mutant. This protein is essential for PSII macro-organization and photoprotection (de Bianchi *et al.*, 2011). Its phosphorylation by SERINE/THREONINE-PROTEIN KINASE STN7 in response to high light is required for PSII–light harvesting complex (LHC) disassembly (Fristedt and Vener, 2011), an integral step of the PSII repair mechanism (Nath *et al.*, 2013). Furthermore, chloroplast SIGNAL RECOGNITION PARTICLE 54 (cpSRP54) is involved in sorting proteins of the LHC family, including Lhcb4.2, to the thylakoid membrane (Rutschow *et al.*, 2008). cpSRP54 was found to be up-regulated in WT, but it was not significantly changed in the mutants. The contrasting regulation of Lhcb4.2 and cpSRP54 in WT and the mutants likely indicates the different fates of the PSII–LHC supercomplex. Changes in Lhcb4.2 and cpSRP54 abundances are likely connected to the early response of Arabidopsis to MV-induced oxidative stress, which are hindered in the hypersensitive *fsd1* mutants.

Other proteins differentially regulated in WT plants upon MV treatment are also involved in PSII or thylakoid photoprotection, including PPL-2 (up-regulated; Ishihara *et al.*, 2007; Ifuku *et al*., 2011) and Psb27 (down-regulated; Chen *et al.*, 2006; Wei *et al.*, 2010). Notably, PPL-1 (Che *et al.*, 2020) shows over 6-fold increased abundance compared with the mock control. This unprecedented accumulation points to the importance of PPL-1 for the plant’s early response to MV-induced oxidative stress. OUTER PLASTID ENVELOPE PROTEIN 16-1, also known as NADPH PROTOCHLOROPHYLLIDE OXIDOREDUCTASE A (PORA) translocation pore, is down-regulated in WT and prevents singlet oxygen production (Buhr *et al.*, 2008). In addition, WT also showed down-regulation of VAR2, a protease involved in D1 degradation and repair cycle (Takechi *et al.*, 2000; Kato *et al.*, 2009). This VAR2 forms a complex with VAR1, both contributing to D1 protein degradation and to the PSII repair cycle (Sakamoto *et al.*, 2003). Hence, the differential proteomes of *fsd1* mutants contained some other photoprotective proteins including PHOTOSYSTEM II SUBUNIT P-1, which was down-regulated in *fsd1-2* mutant and is required for normal thylakoid architecture in Arabidopsis (Yi *et al.*, 2009), and THYLAKOID LUMEN 18.3 kDa PROTEIN involved in PSII repair cycle (Sirpiö *et al.*, 2007; Järvi *et al.*, 2016). CP43 undergoes repair during photodamage (Nelson *et al*., 2014) and regulates the accessibility of D1 protein to proteolysis by FtsH proteases (Krynická *et al.*, 2015). The abundance of this protein was up-regulated after MV treatment in *fsd1-2* mutant. In summary, these results indicate that FSD1 deficiency is associated with defects in LHC disassembly, but the processes connected to PSII photorepair show a pattern similar to WT.

Unlike in WT, two PSI components, PSI-F and PSI-H1, were up-regulated in *fsd1* mutants (Fig. 2). PSI-F is essential for photoautotrophic growth and contributes to antenna function (Haldrup *et al.*, 2000). This indicates an early alteration of PSI subunits in the sensitive mutant line. Interestingly, PSI subunits were affected also after longer MV treatment in the mutants (Melicher *et al.*, 2022a), further supporting the importance of FSD1 for PSI integrity.

Remarkably, the abundances of chloroplastic chaperones, such as CASEIN LYTIC PROTEINASE B3 (Parcerisa *et al*., 2020), CYCLIN DELTA-3, CHAPERONIN-60 ALPHA, and chloroplastic HSP70 (Kessler and Schnell, 2009), were changed only in *fsd1* mutants. CYCLIN DELTA-3 functions downstream of CHAPERONIN-60 in the assembly of chloroplast ATP SYNTHASE COUPLING FACTOR 1 (Mao *et al.*, 2015). These results suggest that modulations in abundance of chloroplastic chaperones represent an early response to MV-induced oxidative stress in the mutant hypersensitive lines.

FSD1 deficiency caused modulation of metabolic protein abundance in chloroplasts, cytoplasm, mitochondria, and peroxisomes already after 30 min of MV treatment (Fig. 2). This implies that missing FSD1 and the associated deregulation of photosynthesis resulted in the transduction of signals to metabolic pathways occurring in other subcellular locations. The redox state of the chloroplast is transduced to the cellular environment via three major redox regulatory hubs, including the ferredoxin–thioredoxin system, the NADPH–NADPH THIOREDOXIN REDUCTASE C (NTRC) system and the glutathione–glutaredoxin system (Dietz and Hell, 2015). Of these systems, NTRC showed an increased abundance in WT. Given that the abundance of metabolic proteins remained almost unaltered in WT, we hypothesize that the change in NTRC abundance contributed to retaining the metabolic homeostasis in WT.

On the other hand, the altered metabolic homeostasis in *fsd1* mutants might arise from a dramatic reduction in the abundance of cytosolic NAD-DEPENDENT MALATE DEHYDROGENASE 1 (MDH1). MDHs and malate/oxaloacetate translocators enable an indirect transfer of reducing equivalents between different subcellular compartments in plant cells (Selinski and Scheibe, 2019). MDH1 is active under reducing conditions, and its homodimerization through Cys330 disulfide formation protects the protein from overoxidation (Huang *et al.*, 2018).

In summary, we provide a specific set of chloroplastic and metabolic proteins showing fast response to MV-induced oxidative stress in plants.

### PATL is involved in plant response to methyl viologen-induced oxidative stress

PATLs are phosphoinositide binding/transfer proteins containing a SEC14 (lipid binding/transfer) and Golgi dynamics (GOLD) domain. The Arabidopsis *PATL* gene family consists of six isoforms (*PATL1–6*). PATL proteins exert a high degree of homology in their amino acid sequence, but with variable N-terminus. PATL1, PATL2, and PATL4 show high levels of similarity, because their N-terminus contains numerous EEK repeats (reminiscent of those found in the neurofilament triplet H proteins) and a coiled-coil, a common protein oligomerization/folding motif (Peterman *et al.*, 2004). PATLs are localized at the plasma membrane and cell plate during cytokinesis (Peterman *et al.*, 2004; Tejos *et al.*, 2018). PATL1 is also localized in brefeldin A-sensitive endosomal compartments (Zhou *et al.*, 2018), indicating its participation in the vesicular trafficking pathway. PATLs, including PATL4, are expressed in meristematic cells, including root apical meristem, lateral root primordia and cells entering a differentiation program (Tejos *et al.*, 2018). In cotyledons, PATL4 localizes mainly to precursors of stomata and subcellularly to plasma membrane (Tejos *et al.*, 2018).

PATLs have diverse functions. For example, PATL1 interacts with SALT-OVERLY-SENSITIVE 1 (SOS1) and controls its Na^+^/H^+^ antiporter activity, thus negatively affecting Arabidopsis salt stress tolerance (Zhou *et al.*, 2018). PATL1 also regulates plant freezing tolerance by interaction with Arabidopsis CALMODULIN 4 (Chu *et al.*, 2018). PATL2 is phosphorylated by MITOGEN-ACTIVATED PROTEIN KINASE 4 and contributes to the membrane regeneration during cell plate formation (Suzuki *et al.*, 2016). Additionally, PATL2 affects Fe^2+^ acquisition responses by interaction with IRON-REGULATED TRANSPORTER1 (IRT1). Arabidopsis *patl2* knockout mutants show elevated lipid peroxidation and reduced levels of α-tocopherol, and PATL2 binds α-tocopherol through its Sec14 domain *in vitro*. It was suggested that PATL2 might present α-tocopherol at the membrane close to IRT1 and protect the membrane from detrimental Fe^2+^ effects. PATL2, in addition, may bind several proteins responsive to oxidative stress, and thus it might attenuate cellular ROS stress or contribute to ROS signaling at the plasma membrane of root cells (Hornbergs *et al.*, 2023).

Involvement of PATLs related to plant oxidative stress in chloroplasts has not been studied so far. Here we detected down-regulation of PATL4 preferentially enriched in plasma membrane of stomata precursors, after a short-term MV treatment, indicating its high sensitivity to ROS generated in chloroplasts (Fig. 4). The absence of *PATL4* expression in mutants confers their higher tolerance to oxidative stress caused by MV (Fig. 5). It is known that MV negatively affects stomatal conductance and transpiration rate leading to intercellular CO_2_ accumulation (Caverzan *et al.*, 2014; Ozfidan-Konakci *et al.*, 2023). Elevated CO_2_ and its conversion to bicarbonate (HCO_3_^–^) leads to induction of anion efflux through the guard cell plasma membrane, positively affecting stomatal closure, mediated by Ca^2+^ signaling (McAinsh *et al.*, 1996; Ehonen *et al.*, 2019). PLASMA MEMBRANE INTRINSIC PROTEIN 2;1 (PIP2;1), a potential interaction partner of PATL4 (Bellati *et al.*, 2016), functions as a membrane channel for CO_2_ (Wang *et al.*, 2016). Therefore, considering the lower stomatal aperture of *patl4* mutants (Fig. 6), it is possible that PATL4 regulates stomatal movements by modulation of CO_2_ transport in guard cells during MV treatment. Notably, these CO_2_-mediated stomatal movements seem to be ABA-independent (Hsu *et al.*, 2018). Nevertheless, several PATL4 interactors might be involved in ABA-mediated stomatal movements. Thus, PIPs, including the above-mentioned PIP2.1 and PIP1.2 (Bellati *et al.*, 2016), are involved in ABA-mediated stomatal closure by regulating plasma membrane water permeability in guard cells (Grondin *et al.*, 2015). Moreover, PATL4 interacts with SMALL UBIQUITIN-LIKE MODIFIER 1 and 3 (SUM1 and 3), and SUMO CONJUGATING ENZYME 1A (SCE1), all implicated in ABA signaling likely through sumoylation of ABA-related signaling proteins (Lois *et al.*, 2003).

According to our results, differential proteomics on mutants defective in important antioxidant enzyme after short-term treatment with MV proved to be feasible for the identification of novel proteins involved in the plant oxidative stress response. Such proteins found in our study may become valuable targets for potential biotechnological applications and stress tolerance/resistance improvements in crops.

## Supplementary data

The following supplementary data are available at *JXB* online.

Fig. S1. Full scan of the entire original immunoblots presented in Fig. 3A.

Fig. S2. GFP–PTL4 localization in epidermal cells of Arabidopsis petiole and leaf after methyl viologen treatment in the dark.

Fig. S3. Early developmental phenotypes of *patl4* mutants.

Table S1. Summary of differentially regulated proteins found by comparison of methyl viologen-treated and mock-treated Arabidopsis WT.

Table S2. Summary of differentially regulated proteins found by comparison of methyl viologen-treated and mock-treated Arabidopsis *fsd1-1* mutant.

Table S3. Summary of differentially regulated proteins found by comparison of methyl viologen-treated and mock-treated Arabidopsis *fsd1-2* mutant.

Table S4. Summary of differentially regulated proteins found by comparison of methyl viologen-treated Arabidopsis *fsd1-1* mutant with methyl viologen-treated WT.

Table S5. Summary of differentially regulated proteins found by comparison of methyl viologen-treated Arabidopsis *fsd1-2* mutant with methyl viologen-treated WT.

erad363_suppl_Supplementary_Figures_S1-S3_Tables_S1-S5

## Data Availability

The mass spectrometry proteomics data have been deposited at the ProteomeXchange Consortium via the PRIDE (Perez-Riverol *et al.*, 2019) partner repository with the dataset identifier PXD038495.
