# Supplementary material for: Methyl viologen-induced changes in the Arabidopsis proteome implicate PATELLIN 4 in oxidative stress responses
Source: J Exp Bot. 2023 Sep 20;75(1):405–21. doi: 10.1093/jxb/erad363 (PMC10735431; doi:10.1093/jxb/erad363)
Supplement: erad363_suppl_Supplementary_Figures_S1-S3_Tables_S1-S5 [file erad363_suppl_supplementary_figures_s1-s3_tables_s1-s5.pdf]

Supplemental Figures and Tables

Arabidopsis early proteome-wide responses to methyl viologen involve PATELLIN 4 implicated in oxidative stress

Pavol Melicher[1], Petr Dvořák[1], Jan Řehák[1], Tibor Pechan[2], Olga Šamajová[1], Jozef Šamaj[1], Tomáš Takáč[1*]

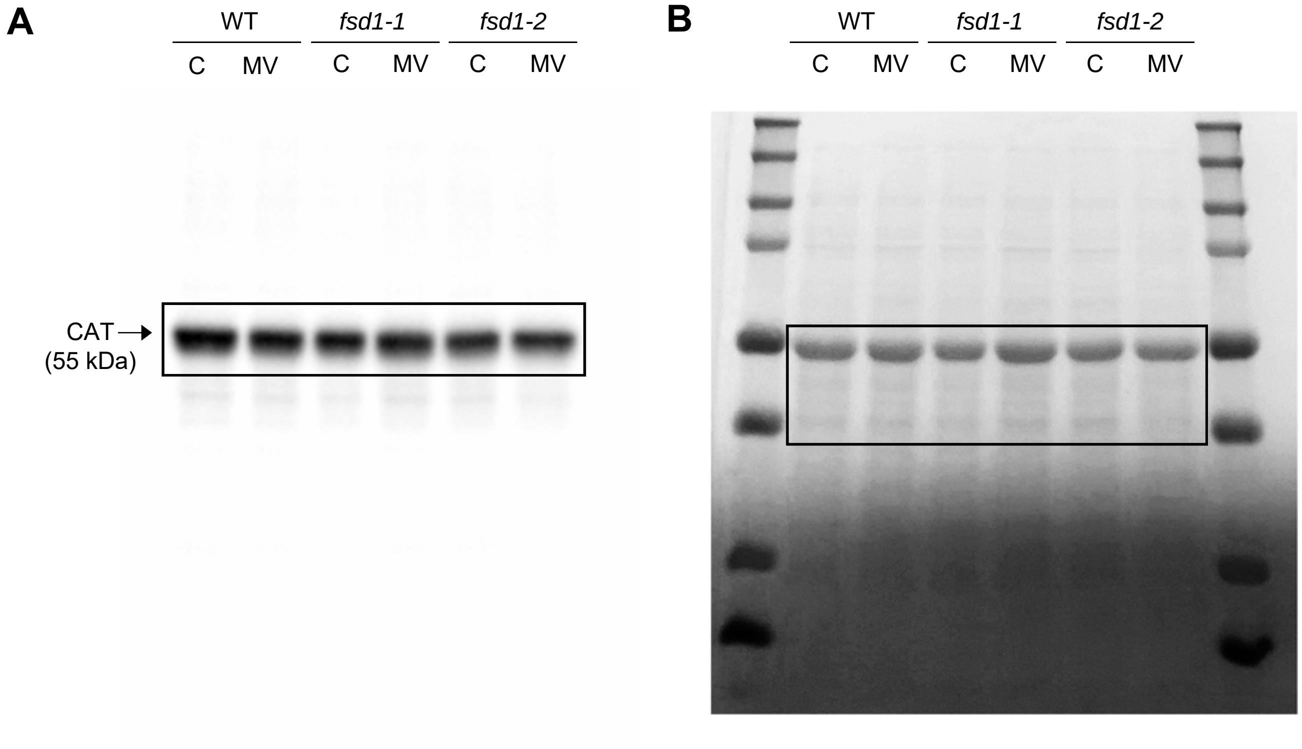

Figure S1. Full scan of the entire original immunoblots presented in Figure 3A. (A) Entire membrane with chemiluminiscent signal observed after probing with anti-catalase antibody. (B) Respective controls of protein loading as visualized by Ponceau S staining. The highlighted regions show the sections presented in Figure 3.

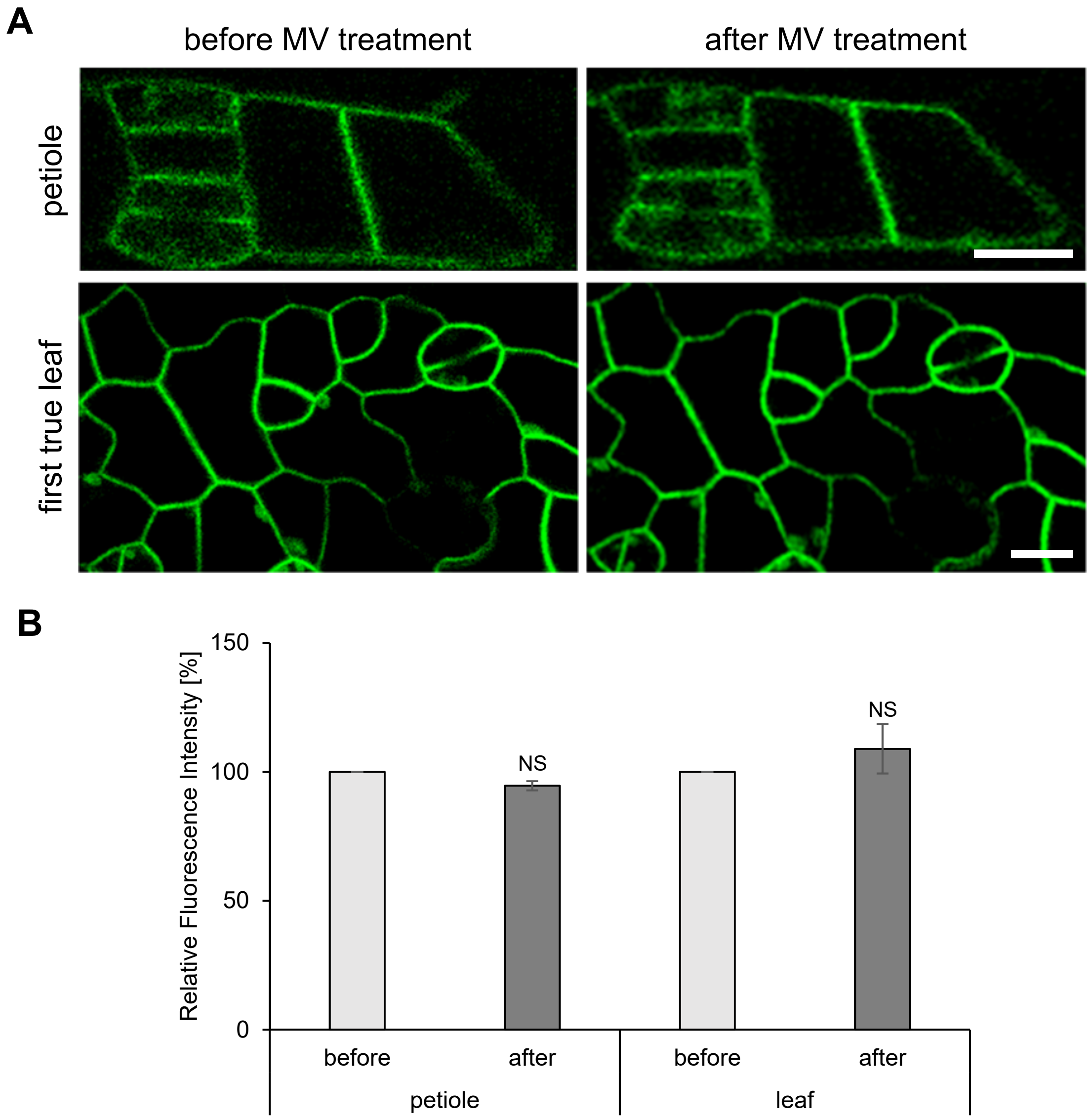

Figure S2. GFP-PTL4 localization in epidermal cells of Arabidopsis petiole and leaf after methyl viologen treatment (MV) in the dark revealed by confocal laser scanning microscopy. (A) Plasma membrane localization of GFP-PTL4 in epidermal cells of petiole and first true leaf before and after 30 min treatment with MV in the dark. (B) Semiquantitative analysis of plasma membrane GFP-PTL4 fluorescence intensity of petiole and leaf epidermal cells before and after 30 min treatment with MV in the dark. NS indicate non statistical significant difference between treated and not treated cells. Error bars show ± standard deviation (SD). Bar = 10 µm.

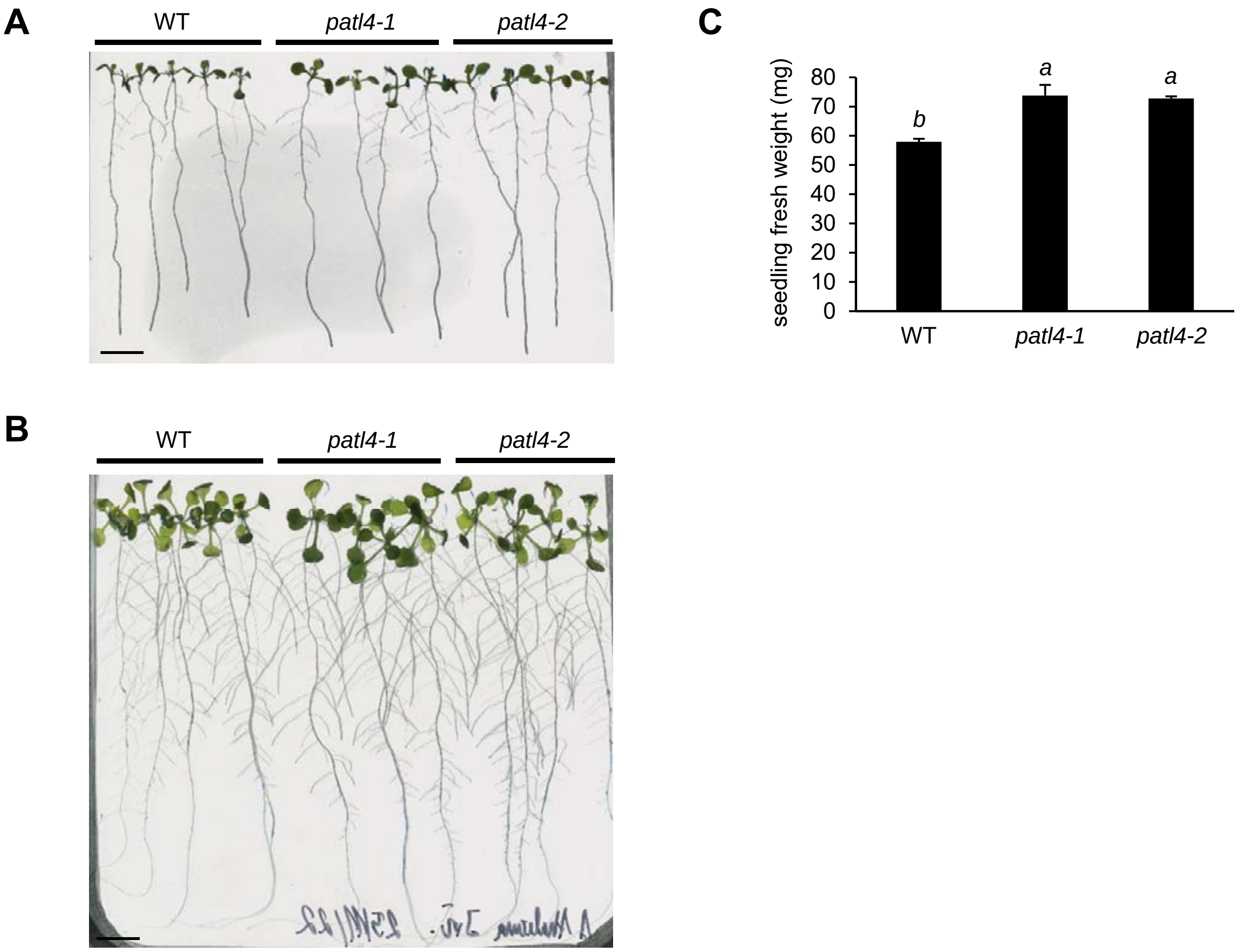

Figure S3. Early developmental phenotypes of *patl4* mutants. Representative image of 7-days-old (A) and 11-days-old (B) WT, *patl4-1* and *patl4-2* mutants. (C) Quantification of fresh weight of 14-day-old seedlings. Lowercase letters above the columns indicate statistical significance between lines according to one-way ANOVA with post hoc Tukey HSD test ($P < 0.05$; mean ±SD. 15 to 19 seedlings per n; n = 6). Bar = 1 cm.

Table S1. Summary of differentialy regulated proteins found by comparison of methyl viologen (MV) - treated and mock-treated Arabidopsis Col-0 (WT). „Unique in C“ means, that the protein was not identified in any of the 4 replicates of the MV treated WT plant samples, but it was detected in at least 3 of the replicates of mock treated WT. „Unique in MV“ means, that the protein was not identified in any of the 4 replicates of the mock - treated WT plant samples, but it was detected in at least 3 of the replicates of MV-treated WT samples. NA = not applicable.

Accession (NCBI)	Localization (Uniprot)	Description	Abundance ratio (MV treatment vs mock treatment)	P-value	MV treated WT-mean	MV treated WT--variance	mock treated WT--mean	mock treated WT--variance
NP_190481.1	apoplast	peroxidase CB (PRXCB)	Unique in C	NA	0	0	254070.79	1701171505.27
NP_181372.1	apoplast	peroxidase superfamily protein (PER22)	Unique in MV	NA	1547142.94	68598123989.27	0	0
NP_001185408.1	Cytoplasm	dehydrin family protein (ERD14)	4.71	0.009	8201098.18	1201624143827.1	1741648.69	1466378681148.29
NP_200414.1	Cytoplasm	heat shock protein 81-2 (HSP90.2)	Unique in C	NA	0	0	5644770.57	2396505757481.62
NP_001331015.1	Cytoplasm. Nucleus	general regulatory factor 5 (GRF5)	Unique in C	NA	0	0	1107930.94	475960152106.63
NP_001319513.1	Cytoplasm. Nucleus	ubiquitin 8 (UBQ8)	Unique in MV	NA	589897.735	8287212354.31	0	0
NP_850594.1	ER body	PYK10-binding protein 1 (PBP1)	0.22	0.048	2281053.83	1428770643911.82	10208579.22	25775160805783.5
NP_563687.1	chloroplast	photosystem II family protein (PSB27)	0.12	0.008	2062352.23	3022162887676.47	17536908.58	27183415735874.3
NP_191093.1	chloroplast	PsbP-like protein 1 (PPL-1)	6.56	0.042	1069685.75	127694928469.95	163036.45	2185937663.98
NP_174575.1	chloroplast	plastid ribosomal protein l11 (PRPL11)	0.32	0.0009	495329.43	1949156308.32	1576193.47	242283033.08
NP_850903.1	chloroplast	ribosomal protein S1 (RPS1)	2.04	0.004	10511547.42	389068382724.35	5161115.34	3544138571519.71
NP_001323509.1	chloroplast	FtsH extracellular protease family (VAR2)	0.32	0.049	907702.85	425397397225.28	2852310.50	480875915679.87
NP_196079.1	chloroplast	sulfite reductase (AtSIR)	Unique in MV	NA	3910500.74	101916580738.91	0	0
NP_187506.1	chloroplast	light harvesting complex photosystem II (Lhcb4.2)	Unique in C	NA	0	0	637639.63	52665051478.72
NP_565906.1	chloroplast	PsbP-like protein 2 (PPL-2)	Unique in MV	NA	408600.36	3550781660.76	0	0
NP_180456.1	chloroplast	outer plastid envelope protein 16-1 (AtOep16-1)	Unique in C	NA	0	0	835104.81	506755271916.03
NP_196014.1	chloroplast	chloroplast signal recognition particle 54 kDa subunit (cpSRP54)	Unique in MV	NA	1542983.37	1576033493094.89	0	0
NP_001326410.1	chloroplast	plastid-lipid associated protein PAP / fibrillin family protein (FBN3B)	Unique in MV	NA	1815041.91	624322564425.94	0	0
NP_565954.1	chloroplast	NADPH-dependent thioredoxin reductase C (NTRC)	Unique in MV	NA	187883.94	812221586.50	0	0
NP_190941.1	Nucleus	histone superfamily protein (H4)	10.84	0.045	16531127.47	20480801584546.2	1524890.08	1735938545629.92
NP_177360.1	plasma membrane	patellin 1 (PATL1)	0.42	0.024	857231.59	162051453687.32	2057164.63	488764721701.69
NP_001031119.1	plasma membrane	Sec14p-like phosphatidylinositol transfer family protein (PATL4)	Unique in C	NA	150861.18	2814179284.63	0	0
NP_563991.1	unknown	nuclear protein	Unique in C	NA	0	0	1416293.06	392588805496.87

Table S2. Summary of differentialy regulated proteins found by comparison of methyl viologen (MV) - treated and mock-treated Arabidopsis *fsd1-1* mutant. „Unique in C" means, that the protein was not identified in any of the 4 replicates of the MV - treated *fsd1-1* mutant plant samples, but it was detected in at least 3 of the replicates of mock treated *fsd1-1*. „Unique in MV" means, that the protein was not identified in any of the 4 replicates of the mock - treated *fsd1-1* mutant plant samples, but it was detected in at least 3 of the replicates of MV treated *fsd1-1*. NA = not applicable.

Accession (NCBI)	Localization (Uniprot)	Description	Abundance ratio (MV treatment vs mock treatment)	P-value	MV treated *fsd1-1*-mean	MV treated *fsd1-1*-variance	Mock-treated *fsd1-1*-mean	Mock-treated *fsd1-1*-variance
NP_001030792.1	cell wall	UDP-glucose 6-dehydrogenase family protein (UGD2)	Unique in MV	NA	705531.37	46066377982.11	0	0
NP_001326523.1	cytoplasm	calmodulin 7 (CAM7)	0.22	0.009	1114913.43	594779463847.13	5098868.87	1601541033898.14
NP_171936.1	cytoplasm	lactate/malate dehydrogenase family protein (MDH1)	0.13	0.032	4536114.07	18823436543872.5	34014462.29	231483521970708
NP_566768.1	cytoplasm	glyoxylate reductase 1 (GLYR1)	0.30	0.023	451741.70	59442373035.81	1486234.63	93767979400.15
NP_568988.2	cytoplasm	aldolase-type TIM barrel family protein (ATBF)	Unique in MV	NA	416945.90	5450255643.06	0	0
NP_179760.1	cytoplasm. nucleus	cold. circadian rhythm. and rna binding 2 (GRP7)	1.75	0.011	24017644.46	9011162466080.32	13726594.49	6919399882858.22
NP_001327587.1	cytoplasm. nucleus	general regulatory factor 7 (GRF7)	Unique in C	NA	0	0	1005343.83	12208249431.76
NP_174486.1	chloroplast	phosphoribulokinase (AtPRK)	0.13	0.004	7209743.71	80843999010612.7	54987309.71	119848454187080
NP_191320.1	chloroplast	adenylosuccinate synthase (AtPUR11)	6.56	0.008	3325706	113934853377.21	507297.35	17124002759
NP_189221.1	chloroplast	ribosomal protein L15 (RPL15)	0.53	0.009	784146.18	6520532135.47	1474569.83	2475323807.82
NP_180367.1	chloroplast	chaperonin-60alpha (CPN60a)	0.55	0.046	38180929.17	278334744269118	69878131.56	93941238744407.7
NP_191873.1	chloroplast	chloroplast stem-loop binding protein of 41 kDa (CSP41A)	18.03	0.005	8702390.64	664864889025.24	482759.41	16225836289.00
NP_199802.1	chloroplast	chloroplast heat shock protein 70-2 (cpHSP70-2)	2.65	0.020	6150351.34	394553627153.71	2322149.57	2807753272777.58
NP_564667.1	chloroplast	thylakoid lumen 18.3 kDa protein (TLP18.3)	0.43	0.010	1015822	9854113400.70	2358418.83	171095414781.55
NP_566100.2	chloroplast	CP12 domain-containing protein 1 (CP12-1)	Unique in C	NA	0	0	1050839.47	559082477743.05
NP_568604.1	chloroplast	FtsH extracellular protease family (VAR1)	Unique in C	NA	0	0	2010674.63	3156018878841.96
NP_001190079.1	chloroplast	chloroplast RNA-binding protein 29 (CP29A)	Unique in MV	NA	2248636.64	993999595409.54	0	0
NP_196447.1	mitochondria	succinyl-CoA ligase. alpha subunit (SUCLG1)	Unique in C	NA	0	0	2041838.03	42629121385.37

Table S3. Summary of differentialy regulated proteins found by comparison of methyl viologen (MV) - treated and mock-treated Arabidopsis *fsd1-2* mutant. „Unique in C“ means, that the protein was not identified in any of the 4 replicates of the MV - treated *fsd1-2* mutant plant samples, but was detected in at least 3 of the replicates of mock treated *fsd1-2*. „Unique in MV“ means, that the protein was not identified in any of the 4 replicates of the mock - treated *fsd1-2* mutant plant samples, but it was detected in at least 3 of the replicates of MV - treated *fsd1-1*. NA = not applicable

.

Accession (NCBI)	Localization (Uniprot)	Description	Abundance ratio (MV treatment vs mock treatment)	P-value	MV-treated *fsd1-2*-mean	MV-treated *fsd1-2*-variance	Mock-treated *fsd1-2*-mean	Mock-treated *fsd1-2*-variance
NP_568479.1	apoplast	glucoside glucohydrolase 2 (Beta glucosidase 37; BGLU37)	Unique in MV	NA	1048229.75	162226218367.83	0	0
NP_188048.1	cell wall	pectin methylesterase 3 (PME3)	Unique in MV	NA	783544.73	20761342077.50	0	0
NP_849993.5	cytoplasm	putative N-acetyl-gamma-glutamyl-phosphate reductase (AtNAGPR)	Unique in MV	NA	1434351.06	748677862317.77	0	0
NP_564167.1	cytoplasm. nucleus	general regulatory factor 10 (GFR10)	2.28	0.0008	784415.31	2578048598.70	343665.51	3321393848.54
NP_200637.1	cytoplasm. nucleus	regulatory particle triple-A ATPase 3 (RPT3)	0.55	0.045	472042.99	8094390645.99	853460.08	19530756165.80
NP_849607.1	cytosol	ascorbate peroxidase 1 (APX1)	0.33	0.049	4607235.34	8323602448117.41	13771819.48	47267330061764.40
NP_001154674.1	cytosol	monodehydroascorbate reductase 1 (MDHAR 1)	2.53	0.035	2104202.95	195336075285.11	831083.46	50878588074.51
NP_200412.1	cytosol	heat shock protein 81-3 (HSP70.3)	Unique in MV	NA	3364755.90	6899212925198.91	0	0
NP_001154684.1	chloroplast	rotamase CYP 4 (CYP4)	2.97	0.002	128849487.53	886886811639728	43350572.75	248347206213918
NP_190442.1	chloroplast	glutamate-1-semialdehyde 2.1-aminomutase 2 (GSA2)	12.50	0.034	1154538.19	328752941182.55	92381.48	231292958.84
NP_568049.1	chloroplast	fructose-bisphosphate aldolase 2 (FBA2)	0.36	0.016	15058975.69	26420601423366.90	42293138.79	247655959206478
NP_176015.1	chloroplast	phosphoglycerate kinase family protein (PGK2)	3.21	0.048	1127428.68	364772890144.42	351688.18	27456610586.46
NP_195146.1	chloroplast	D-3-phosphoglycerate dehydrogenase (PGDH1)	0.40	0.003	141227.24	280737727.68	356686.68	6462093.31
NP_173786.1	chloroplast	oxidoreductase. zinc-binding dehydrogenase family protein (AOR)	2.13	0.004	2518709.66	41905259396.20	1184323.18	31911693265.85
NP_567265.1	chloroplast	ATPase. F1 complex. gamma subunit protein (ATPC1)	2.54	0.025	47165644.46	154372385595478	18538341.80	218332821102660
NP_564010.1	chloroplast	uridylyltransferase-like protein (ACR11)	4.40	0.010	13089049.76	20714103816001.60	2973202.57	1688932772542.85
NP_174418.1	chloroplast	photosystem I subunit F (PSAF)	2.07	0.012	31367361.78	47541109183758.50	15158164.42	35696487088818.20
NP_172153.1	chloroplast	photosystem II subunit P-1 (PSBP-1)	0.30	0.029	25352114.37	250140929326118	83359281.44	1407008786661870.00
NP_194159.1	chloroplast	chloroplast heat shock protein 70-1 (cpHSP70)	0.38	0.008	3365789.97	4019865943730.84	8823495.80	936608703209.23
NP_196846.1	chloroplast	aldolase-type TIM barrel family protein (TRA2)	Unique in MV	NA	0	0	458651.21	3968222180.44
NP_181837.1	chloroplast	aconitase/3-isopropylmalate dehydratase protein (SSU1)	Unique in MV	NA	389270.11	4140732749.09	0	0
NP_197143.1	chloroplast	chloroplastic acetylcoenzyme A carboxylase 1 (CAC1A)	Unique in MV	NA	56958.80	135111278.68	0	0
NP_176896.1	chloroplast	glyoxalase/Bleomycin resistance protein/Dioxygenase superfamily protein (ATGLYI6. GLYOXALASEI 6)	Unique in MV	NA	1339716.27	7908904604.15	0	0
NP_201210.1	chloroplast	glutamate tRNA synthetase (OVA3)	Unique in MV	NA	242767.16	1563204527.53	0	0
NP_173025.1	chloroplast	cobalamin biosynthesis CobW-like protein (CobWLP)	Unique in MV	NA	4047408.10	13752207836720.50	0	0
NP_198202.1	chloroplast	rubisco accumulation factor-like protein (RAFLP)	Unique in MV	NA	330773.24	29896857905.46	0	0
NP_001190908.1	chloroplast	cyclin delta-3 (PROTEIN IN CHLOROPLAST ATPASE BIOGENESIS. PAB)	Unique in MV	NA	671776.43	10240279493.59	0	0
NP_188235.1	chloroplast	photosystem I subunit H-1 (PSAH-1)	Unique in MV	NA	9072883.44	926765954527.44	0	0
NP_051055.1	chloroplast	photosystem II 44 kDa protein (CP43)	Unique in MV	NA	964342.78	138096145.68	0	0
NP_187506.1	chloroplast	light harvesting complex photosystem II (LHCb4.2)	Unique in MV	NA	3134087.88	12663211691677.90	0	0
NP_180936.1	chloroplast	ribosomal protein S5 family protein (PRPS5)	Unique in MV	NA	5240639.53	24457874910786.50	0	0
NP_001319848.1	chloroplast	translocon at the outer envelope membrane of chloroplasts 159 (TOC159)	Unique in MV	NA	61330.46	48807552.10	0	0
NP_568314.1	chloroplast	casein lytic proteinase B3 (CLPB3)	Unique in MV	NA	309194.00	7915701280.44	0	0
NP_196079.1	chloroplast	sulfite reductase (SIR)	Unique in C	NA	0	0	3912689.38	12490420593064.20
NP_177239.1	chloroplast	phosphoglucomutase. putative / glucose phosphomutase (PGM)	Unique in MV	NA	111978.63	92655308.89	0	0
NP_568289.3	chloroplast	elongation factor family protein (SVR3)	Unique in MV	NA	503674.67	178542154612.83	0	0
NP_850203.1	mitochondria	heat shock protein 60-2 (HSP60-2)	3.19	0.024	487406.15	11088509011.68	152694.31	943623108.01
NP_186777.1	mitochondria	voltage dependent anion channel 1 (VDAC1)	4,23	0.033	2058388.25	129192698833.92	486252.89	44173873603.91
NP_201477.1	mitochondria	succinate dehydrogenase 1-1 (SDH1-1)	Unique in MV	NA	603186.40	179392987182.02	0	0
NP_196647.1	mitochondria	cystathionine beta-synthase (CBS) family protein (CBSX3)	Unique in MV	NA	263156.44	3207547322.81	0	0
NP_001031263.1	peroxisome	alanine-2-oxoglutarate aminotransferase 2 (AOAT2)	Unique in MV	NA	573511.36	84073940281.55	0	0
NP_849951.1	peroxisome	alanine:glyoxylate aminotransferase (AGT1)	Unique in MV	NA	747073.57	117259733913.15	0	0
NP_179863.1	peroxisome	peroxisomal NAD-malate dehydrogenase 1 (PMDH1)	Unique in MV	NA	173778.61	1890375004.46	0	0
NP_188060.1	peroxisomes	Aldolase-type TIM barrel family protein (GOX1)	14,53	0.036	16081867.95	25526962659693.40	1106667.77	362718298230.57
NP_001328611.1	peroxisomes	catalase 2 (CAT2)	2,75	0.027	43055052.06	123748264950274	15645874.28	232173526906243
NP_001154535.1	ribosome	60S acidic ribosomal protein family (RPP2A)	Unique in C	NA	0	0	903509.73	725765552496.71
NP_850411.1	ribosome	ribosomal protein L30/L7 family protein (RPL7C)	Unique in C	NA	0	0	346189.66	10821532319.40
NP_173045.1	ribosome	ribosomal protein L7Ae/L30e/S12e/Gadd45 family protein (RPS12A)	Unique in MV	NA	425437.67	10116852938.47	0	0
NP_197024.1	ribosome	ribosomal protein S4 (RPS9B)	Unique in MV	NA	3499057.14	516659734361.91	0	0
NP_186857.1	ribosome	ribosomal protein S19e family protein (RPS19A)	Unique in MV	NA	5082431.43	12257078403940	0	0
NP_564798.1	vacuole	glycosyl hydrolases family 32 protein (vacuolar invertase 1; VI1)	Unique in MV	NA	3171211.77	520175163831.73	0	0
NP_001185204.1	vacuole	beta glucosidase 18 (BGLU18)	Unique in MV	NA	382790.12	3959477136.69	0	0

Table S4. Summary of differentialy regulated proteins found by comparison of methyl viologen (MV) - treated Arabidopsis *fsd1-1* mutant with MV - treated Col-0 (WT). „Unique in WT“ means, that the protein was not identified in any of the 4 replicates of the MV- treated *fsd1-1* mutant plant samples, but it was detected in at least 3 of the replicates of MV – treated WT. „Unique in *fsd1-1*“ means, that the protein was not identified in any of the 4 replicates of the MV - treated WT plant samples, but it was detected in at least 3 of the replicates of MV - treated *fsd1-1*. NA = not applicable

Accession (NCBI)	Localization (Uniprot)	Description	Abundance ratio (MV treated *fsd1-1* vs WT)	P-value	MV-treated *fsd1-1*-mean	MV-treated *fsd1-1*-variance	MV-treated WT mean	MV-treated WT variance
NP_563851.1	apoplast	Eukaryotic aspartyl protease family protein (AED3)	0.27	0.038	794502.64	268372904722.51	2908184.15	95469506184.65
NP_850759.1	cytoplasm	Aldolase superfamily protein (FBA4)	Unique in WT	NA	773768.57	244075379933.75	0	0
NP_849577.1	cytoplasm	S-adenosylmethionine synthetase 1 (SAM1)	Unique in *fsd1-1*	NA	1175213.20	9054077631.84	0	0
NP_198893.1	cytoplasm, mitochondria, nucleus	prohibitin 3 (PHB3)	Unique in *fsd1-1*	NA	172472.54	232555.06	0	0
NP_191104.1	cytoplasm, mitochondria	triosephosphate isomerase (CTIMC)	0.35	0.027	5780749.62	15046134870118.7	16379497.12	38455601872232
NP_194240.1	chloroplast	Fe superoxide dismutase 1 (FSD1)	Unique in WT	NA	0	0	3395582.52	3157181811883.12
NP_189215.1	chloroplast	2-oxoacid dehydrogenases acyltransferase family protein (LTA2)	3.54	0.011	1966048.17	188269323752.42	556063.45	17256393099.92
NP_194214.2	chloroplast	arginosuccinate synthase family	0.22	0.007	103537.11	4347559605	470113.02	1801591729.13
NP_194193.2	chloroplast	phosphoglucose isomerase 1 (PGI1)	0.79	0.014	225123.49	4528059.04	286079.60	102174477.30
NP_564010.1	chloroplast	uridylyltransferase-like protein (ACR11)	0.37	0.025	5068330.30	9387921086508.9	13875718.02	9635808152321.22
NP_174703.1	chloroplast	2-oxoacid dehydrogenases acyltransferase family protein (EMB3003)	0.70	0.051	373850.04	4810711059.84	536857.45	24486111.34
NP_051065.1	chloroplast	ATP synthase CF1 epsilon subunit (atpE)	0.20	0.026	627837.81	158095841468.84	3164482.15	601704818605.58
NP_188235.1	chloroplast	photosystem I subunit H-1 (PSAH1)	23.82	0.003	10193203.37	487213677276.73	427907.48	33510248274.83
NP_201209.1	chloroplast	photosystem I reaction center subunit PSI-N, chloroplast, putative / PSI-N, putative (PSAN)	0.23	0.034	1894248.52	1217911010328.14	8213367.80	10800122804748.1
NP_051067.1	chloroplast	ribulose-1,5-bisphosphate carboxylase/oxygenase large subunit (RbcL)	1.26	0.002	4044791305.17	2,22831E+16	3215451652.8 7	8018838316782916 3
NP_565786.1	chloroplast	photosystem II light harvesting complex protein B1B2 (Lhb1B2)	0.47	0.023	1955790.06	417755221734.07	4137017.45	1633460610426.06
NP_001318614.1	chloroplast	chaperonin 20 (CPN20)	0.53	0.014	16060145.02	30873241916197.1	30409049.73	21587087003222.4
NP_001319848.1	chloroplast	translocon at the outer envelope membrane of chloroplasts 159 (TOC159)	Unique in WT	NA	0	0	81267.77	41185112.32
NP_001322308.1	chloroplast	chloroplast RNA binding protein (CRB)	0.50	0.041	20812194.29	33615032246991.9	41833627.04	145540116816418
NP_850903.1	chloroplast	ribosomal protein S1 (RPS1)	0.56	0.003	6406956.68	1811382762223.27	11481481.75	464182231260.40
NP_565109.1	chloroplast	Ribosomal protein L31 (RPL31)	0.20	0.013	1926785.03	2086015308663.23	9414555.82	3130520863518.59
NP_173431.1	chloroplast	ferredoxin-NADP[+]-oxidoreductase 2 (LFNR2)	Unique in *fsd1-1*	NA	6131683.32	803073178141.72	0	0
NP_565954.1	chloroplast	NADPH-dependent thioredoxin reductase C (NTRC)	Unique in WT	NA	0	0	205220.60	969029725.98
NP_850504.1	chloroplast	Ribosomal protein S7e family protein (RPS7B)	Unique in *fsd1-1*	NA	340071.90	458675463.78	0	0
NP_001332670.1	chloroplast, cytoplasm	6-phosphogluconate dehydrogenase family protein (PGD3)	0.35	0.0412	142047.27	598110158.11	401796.72	5332674940.72
NP_001184945.1	chloroplast, mitochondria	ATP-dependent caseinolytic (Clp) protease/crotonase family protein	Unique in *fsd1-1*	NA	821232.47	1169196747.89	0	0
NP_568479.1	chloroplast, vacuole, peroxisome, ribosome	glucoside glucohydrolase 2 (TGG2)	Unique in WT	NA	0	0	779716.81	19977403861.90
NP_186777.1	mitochondria	voltage dependent anion channel 1 (VDAC1)	0.39	0.01	1251396.37	55357908513.31	3248319.76	303865884614.30
NP_175683.1	nucleus	histone H2A protein 9	Unique in *fsd1-1*	NA	275567.15	536105651.50	0	0
NP_564192.2	peroxisome	glutamate:glyoxylate aminotransferase (GGAT1)	0.26	0.012	318200.96	2441698393.09	1206720.98	87257323801.55
NP_197056.1	plasmodesma	Ribosomal protein S19e family protein (RPS19B)	Unique in WT	NA	0	0	14084472.21	35579350262736.2
NP_179393.1	ribosome	Ribosomal protein L2 family (RPL8A)	Unique in *fsd1-1*	NA	1049787.01	658837899535.38	0	0
NP_568988.2	unknown	Aldolase-type TIM barrel family protein	Unique in *fsd1-1*	NA	315508.20	3120892139.48	0	0
NP_179760.1	unknown	cold, circadian rhythm, and rna binding 2	2.28	0.032	18174453.25	5159916882757.09	7975371.96	24794297752673.5
NP_849650.1	unknown	Vacuolar calcium-binding protein-like protein	Unique in *fsd1-1*	NA	336314.97	6564360801.32	0	0

Table S5. Summary of differentialy regulated proteins found by comparison of methyl viologen (MV) - treated Arabidopsis *fsd1-2* mutant with MV - treated Col-0 (WT). „Unique in WT“ means, that the protein was not identified in any of the 4 replicates of the MV- treated *fsd1-2* mutant plant samples, but it was detected in at least 3 of the replicates of MV – treated WT. „Unique in *fsd1-2*“ means, that the protein was not identified in any of the 4 replicates of the MV - treated WT plant samples, but it was detected in at least 3 of the replicates of MV - treated *fsd1-1*. NA = not applicable

Accession (NCBI)	Localization (Uniprot)	Description	Abundance ratio (MV treated *fsd1-2* vs WT)	P-value	MV-treated *fsd1-2*-mean	MV-treated *fsd1-2*-variance	MV-treated WT mean	MV-treated WT variance
NP_851234.1	cell wall, nucleus, vacuole, Golgi, ER	protein disulfide isomerase-like 1-4	Unique in WT	NA	0	0	1566059.20	77488906545.35
NP_179566.1	cytoplasm	profilin 1 (PRF1)	Unique in *fsd1-2*	NA	3442559.58	4152251853370.13	0	0
NP_565958.1	cytoplasm	villin 2 (VLN2)	Unique in WT	NA	0	0	186623.02	1197397252.58
NP_564167.1	cytoplasm, nucleus	general regulatory factor 10 (GRF10)	2.12	0.026	752546.23	2372823010.43	354675.50	6121903937.01
NP_001319513.1	cytoplasm, nucleus	ubiquitin 8 (UBQ8)	Unique in WT	NA	0	0	611449.08	8903804268.31
NP_194664.1	cytoplasm, nucleus, ER	profilin 2 (PRF2)	Unique in *fsd1-2*	NA	1025404.99	809669179754.79	0	0
NP_568375.2	cytosol, plastid, vacuole	Translation elongation factor EF1B/ribosomal protein S6 family protein (EF1B)	4.35	0.0001	3545725.55	30165930149.33	815516.13	64029442962.87
NP_180873.1	glyoxysome	peroxisomal 3-ketoacyl-CoA thiolase 3 (PED1)	Unique in *fsd1-2*	NA	251100.03	17973987.50	0	0
NP_194240.1	chloroplast	Fe superoxide dismutase 1 (FSD1)	Unique in WT	NA	0	0	3222304.46	2843178700702.05
NP_194474.1	chloroplast	protochlorophyllide oxidoreductase B (PORB)	Unique in *fsd1-2*	NA	158720.80	172820803.27	0	0
NP_181837.1	chloroplast	aconitase/3-isopropylmalate dehydratase protein (SSU1)	0.63	0.038	373454.91	3811109671.10	592164.44	14338808.17
NP_178171.1	chloroplast	isopropylmalate dehydrogenase 2 (IMDH2)	Unique in *fsd1-2*	NA	653863.63	165316120218.65	0	0
NP_201209.1	chloroplast	photosystem I reaction center subunit PSI-N, chloroplast, putative / PSI-N, putative (PSAN)	0.32	0.043	2454716.20	342913135672.71	7794236.04	9725977454909.73
NP_188235.1	chloroplast	photosystem I subunit H-1 (PSAH1)	21.44	0.001	8704272.01	852990740087.19	406071.18	30177427157.23
NP_001318614.1	chloroplast	chaperonin 20 (CPN20)	0.45	0.003	12968846.50	21816912388568.60	28857262.58	19440105016047.80
NP_174575.1	chloroplast	50S ribosomal protein L11 (RPL11)	3.05	0.038	1567756.20	86898257514.78	513425.81	2094179021.32
NP_181092.1	chloroplast	plastid-lipid associated protein PAP / fibrillin family protein	Unique in WT	NA	0	0	2462208.03	4369076454910.6
NP_200333.2	chloroplast	trigger factor type chaperone family protein (TIG)	Unique in WT	NA	0	0	1912733.07	464222728926.20
NP_850479.1	chloroplast, cytoplasm	glutathione S-transferase phi 8 (GSTF8)	19.29	0.001	8321688.09	1837787808351.54	431461.16	4726796576.65
NP_001154684.1	chloroplast, cytosol	rotamase CYP 4 (ROC4)	2.20	0.008	123614614.33	8,16286E+14	56206207.83	3,69498E+14
NP_566570.3	mitochondria	lipoamide dehydrogenase 2 (LPD2)	Unique in *fsd1-2*	NA	557130.42	66236567338.11	0	0
NP_196647.1	mitochondria	cystathionine beta-synthase (CBS) family protein (CBSX3)	Unique in *fsd1-2*	NA	252464.97	2952210481.39	0	0
NP_564337.1	mitochondria	glycyl-tRNA synthetase / glycine-tRNA ligase	Unique in *fsd1-2*	NA	904144.66	101745354181.66	0	0
NP_564192.2	peroxisome	glutamate:glyoxylate aminotransferase (GGAT1)	0.52	0.05	591518.85	15214622014.71	1145141.48	78578992055.21
NP_850106.1	ribosome	60S acidic ribosomal protein family (RPP2B)	13.75	0.042	9893526.25	2,8449E+13	719535.93	110081228626.80
NP_199581.1	ribosome	60S acidic ribosomal protein family (RPP1C)	Unique in *fsd1-2*	NA	2324054.13	1062668243543.23	0	0
NP_001154535.1	ribosome	60S acidic ribosomal protein family (RPP2A)	Unique in WT	NA	0	0	769711.91	231131808973.41
NP_001320652.1	unknown	phosphoenolpyruvate carboxylase family protein	Unique in WT	NA	0	0	1094161.96	435576191023.34
NP_181372.1	vacuole	Peroxidase 22 (PER22)	Unique in WT	NA	0	0	1603666.33	73702017404.69
